# Non-invasive measurement of PD-L1 status and prediction of immunotherapy response using deep learning of PET/CT images

**DOI:** 10.1136/jitc-2020-002118

**Published:** 2021-06-16

**Authors:** Wei Mu, Lei Jiang, Yu Shi, Ilke Tunali, Jhanelle E Gray, Evangelia Katsoulakis, Jie Tian, Robert J Gillies, Matthew B Schabath

**Affiliations:** 1 Department of Cancer Physiology, Moffitt Cancer Center, Tampa, Florida, USA; 2 Department of Nuclear Medicine, Shanghai Pulmonary Hospital, Tongji University School of Medicine, Shanghai, China; 3 Department of Radiology, Shengjing Hospital of China Medical University, Shenyang, China; 4 Department of Thoracic Oncology, Moffitt Cancer Center, Tampa, Florida, USA; 5 Department of Radiation Oncology, James A. Haley Veterans Affairs Medical Center, Tampa, Florida, USA; 6 Beijing Advanced Innovation Center for Big Data-Based Precision Medicine, School of Engineering Medicine, Beihang University, Beijing, China; 7 Institute of Automation, Chinese Academy of Sciences, Beijing, China; 8 Department of Cancer Epidemiology, Moffitt Cancer Center, Tampa, Florida, USA

**Keywords:** tumor biomarkers, immunotherapy

## Abstract

**Background:**

Currently, only a fraction of patients with non-small cell lung cancer (NSCLC) treated with immune checkpoint inhibitors (ICIs) experience a durable clinical benefit (DCB). According to NCCN guidelines, Programmed death-ligand 1 (PD-L1) expression status determined by immunohistochemistry (IHC) of biopsies is the only clinically approved companion biomarker to trigger the use of ICI therapy. Based on prior work showing a relationship between quantitative imaging and gene expression, we hypothesize that quantitative imaging (radiomics) can provide an alternative surrogate for PD-L1 expression status in clinical decision support.

**Methods:**

^18^F-FDG-PET/CT images and clinical data were curated from 697 patients with NSCLC from three institutions and these were analyzed using a small-residual-convolutional-network (SResCNN) to develop a deeply learned score (DLS) to predict the PD-L1 expression status. This developed model was further used to predict DCB, progression-free survival (PFS), and overall survival (OS) in two retrospective and one prospective test cohorts of ICI-treated patients with advanced stage NSCLC.

**Results:**

The PD-L1 DLS significantly discriminated between PD-L1 positive and negative patients (area under receiver operating characteristics curve ≥0.82 in the training, validation, and two external test cohorts). Importantly, the DLS was indistinguishable from IHC-derived PD-L1 status in predicting PFS and OS, suggesting the utility of DLS as a surrogate for IHC. A score generated by combining the DLS with clinical characteristics was able to accurately (C-indexes of 0.70–0.87) predict DCB, PFS, and OS in retrospective training, prospective testing and external validation cohorts.

**Conclusion:**

Hence, we propose DLS as a surrogate or substitute for IHC-determined PD-L1 measurement to guide individual pretherapy decisions pending in larger prospective trials.

## Introduction

The emergence of immune checkpoint inhibitors (ICIs) has revolutionized cancer treatment and improved long-term survival among some patients with advanced stage non-small cell lung cancer (NSCLC), but durable clinical benefit (DCB) is only observed in 20%–50% patients.^1 2^ Because of the complexity and heterogeneity of response, NCCN guidelines recommend treatment based on expression of the checkpoint target, programmed death-ligand 1 (PD-L1), determined by immunohistochemistry (IHC).^3^ Early studies showed that PD-L1 positivity is associated with significantly higher objective response rate, longer progression-free survival (PFS), and longer overall survival (OS).^1 4^ However, measuring PD-L1 by IHC requires surgical or biopsied tumor specimens, which are collected through invasive procedures and associated with risk of morbidities.^5^ Therefore, an alternative non-invasive method of measuring PD-L1 status would have important implications for clinical decision support, especially when tissues are not available or when the IHC fails.^6^

Radiomic analyses of quantitative image features based on shape, size, voxel intensity, and texture are strongly associated with gene and protein expression in NSCLC.^7 8^ Signatures are typically extracted from the intratumoral region, but it is becoming increasingly appreciated that the peritumoral region,^9^ encompassing the tumor-stroma interface, is also informative in predictive models, likely because this region contains information on immune infiltration and stromal inflammation. Intratumoral and peritumoral immune-cell infiltration is necessary for inducing an immunotherapy response. Immune infiltration is associated with expression of cell checkpoint markers including PD-L1,^10^ which is significantly correlated with metabolic rate,^11^ GLUT-1 expression,^12^ pAKT levels,^13^ hypoxia, and acidosis.^14^ These observations suggest that PD-L1 expression might be tractable by radiomics analyses of Fluorine 18 (18F)−fluorodeoxyglucose (FDG)-Positron emission tomography (PET) scans. As a consequence, others have investigated the relationship between FDG-PET and PD-L1 status in NSCLC, but these analyses were limited to a few statistical associations.^15 16^ Our previous study demonstrated the utility of deep learning methods using intratumoral and peritumoral radiomics from PET/CT images to predict epidermal growth factor receptor (EGFR) mutation status, which could be used to support the treatment decisions for EGFR-TKI and other therapies, including ICI, which is generally more effective in EGFR wild-type cancers.^17^

**Figure 1:**
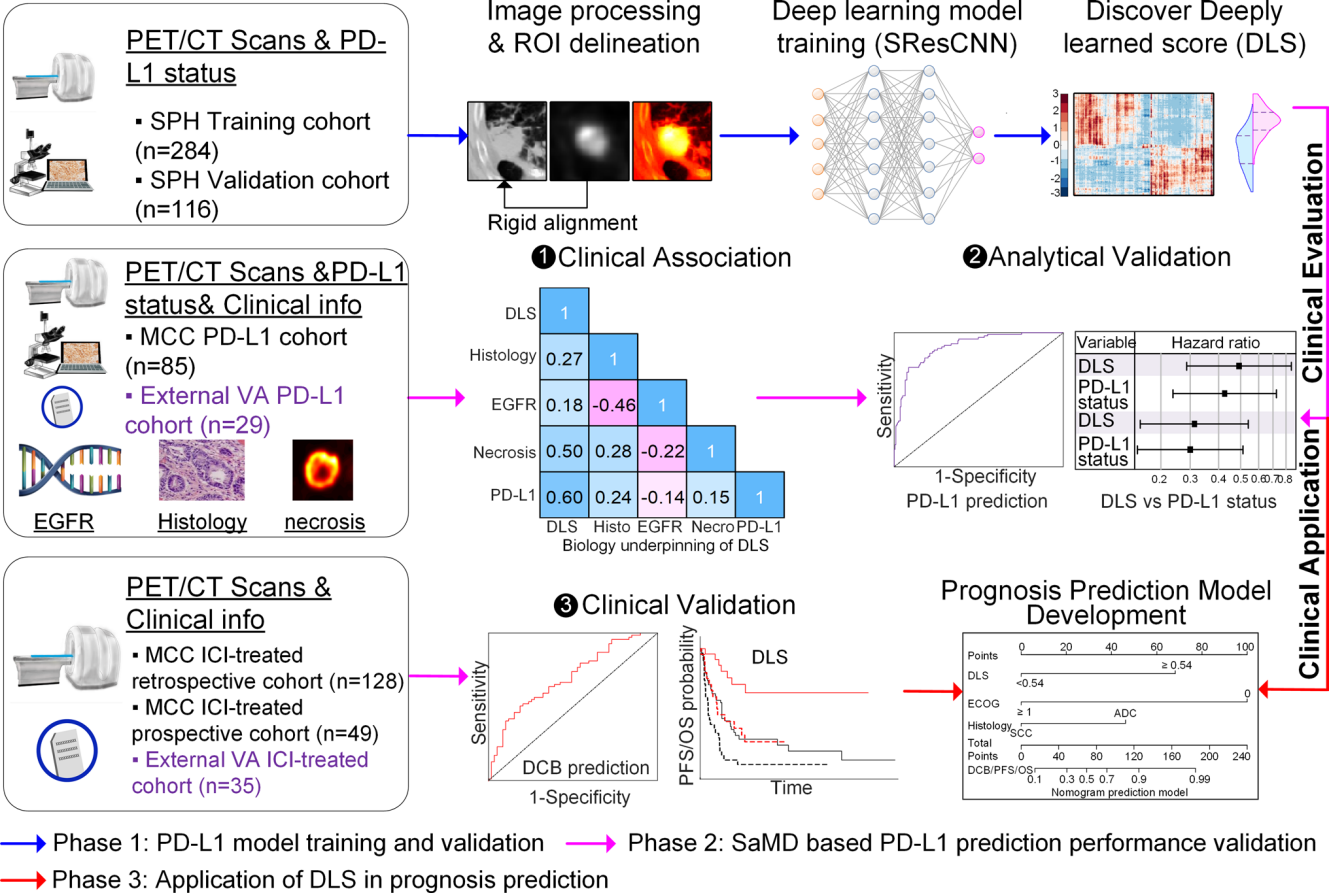
Study design, which contains three main phases. First, the SPH data comprised PD-L1 expression data and the corresponding imaging data was used to train and validate the deeply learned score (DLS). Then, according to FDA SaMD guideline, the DLS was evaluated through the clinical association and analytic validation on the two cohorts (MCC PD-L1 data and external VA PD-L1 data), which had both PD-L1 expression data and clinical follow-up information, as well as the clinical validation on three other cohorts (MCC ICI-treated retrospective, prospective, and external VA ICI-treated cohorts), which had clinical follow-up information. Third, in order to further test the application of DLS in guiding treatment, the well-validated DLS was utilized to develop prognosis prediction models with the MCC ICI-treated retrospective cohort, which was tested with MCC ICI-treated prospective and external VA ICI-treated cohorts. DCB, durable clinical benefit; ICI, immune checkpoint inhibitor; MCC, H Lee Moffitt Cancer Center and Research Institute; ROI, region of interest; SPH, Shanghai Pulmonary Hospital; VA, James A Haley Veterans’ Administration; EGFR, epidermal growth factor receptor; PD-L1, programmed death-ligand 1; FDA, Food and Drug Administration; PET/CT, positron emission tomography/computed tomography.

In the current work, we utilized machine learning to develop and validate a deeply learned score (DLS) to measure PD-L1 expression status non-invasively using pretreatment ^18^F-FDG PET/CT images of a retrospective cohort accrued from Shanghai Pulmonary Hospital (SPH). Then, to validate the DLS in accordance with the FDA guidance document for the Clinical Evaluation of Software as a Medical Device (SaMD),^18^ clinical association analysis for scientific validity, analytic validation analysis for accuracy and reliability, and clinical validation analysis for identifying patients most likely to benefit from ICI treatment were performed using external test cohorts from the H Lee Moffitt Cancer Center and Research Institute (MCC) with both PD-L1 status and clinical follow-up information (ie, MCC PD-L1 cohort) or only with clinical follow-up information (ie, MCC ICI-treated retrospective and prospective cohorts). To determine the potential application for accurate quantitative prognostic prediction, we developed DCB, PFS, and OS prediction models with the derived DLS using the MCC ICI-treated retrospective cohort. The models for all three endpoints were independently tested with the MCC ICI-treated prospective cohort. Finally, an external ICI-treated cohort from a third institution, James A Haley Veterans’ Administration (VA), was used to blindly validate the models mentioned above (details shown in figure 1 and online supplemental figure S1).

10.1136/jitc-2020-002118.supp1 Supplementary data

### Study population

In this multi-institutional study, five cohorts of patients were first accrued from two institutions: SPH, Shanghai, China, and MCC, Tampa, Florida. The detailed inclusion and exclusion criteria are provided in online supplemental figure S1 and methods S1. Among these, the SPH retrospective cohort, which was split into training (N=284) and validation (N=116) cohorts randomly by 71%–29%, and the retrospective MCC cohort with PD-L1 status (N=85) were used for training, validating, and testing the DLS to measure PD-L1 expression status non-invasively; one ICI-treated retrospective cohort (N=128) and one ICI-treated prospective cohort (N=49) were used to validate the prognostic value of the DLS and investigate the association of the DLS and clinical characteristics on the clinical outcomes. Additionally, a sixth cohort (N=35) from the third institution, VA, Tampa, Florida, was curated as an external validation of the DLS and the prognostic models.

The progression of the distinct ICI-treated cohorts used to investigate the association of the DLS and clinical characteristics with the clinical outcome including DCB (PFS >6 months^19^), PFS, and OS, were defined using Response Evaluation Criteria in Solid Tumors (RECIST V.1.1).^20^ The index date for both OS and PFS was the date of initiation of immunotherapy.

The requirement for informed consent was waived by the IRBs, as no personal health information is reported.

### ^18^F-FDG PET/CT imaging

Detailed acquisition parameters for the ^18^F-FDG PET/CT imaging for each cohort are presented in online supplemental table S1. All PET images were converted into standardized uptake value (SUV) units by normalizing the activity concentration to the dosage of ^18^F-FDG injected and the patient’s body weight after decay correction.

### PD-L1 expression by IHC

The detailed information of IHC staining for PD-L1 expression is provided in online supplemental methods S2. For both SPH and MCC PD-L1 cohort, the platform of Dako Link 48 and the antibody of Dako 22C3 were used for PD-L1 staining to quantify the presence of PD-L1. The level of PD-L1 expression was presented as a tumor proportion score (TPS), which is the percentage of viable tumor cells showing membrane PD-L1 staining relative to all viable tumor cells and is given as 0%, 1%–49%, and ≥50%, and PD-L1 positivity was defined as ≥1% of TPS.^21 22^ To compensate for reader bias, all the staining results were reviewed and analyzed by two experienced pathologists who were blinded to each other’s scores and unaware of the patients’ clinical information. When there was discrepancy, the two pathologists would have a mutual discussion to reach a consensus.

### Development of the DLS

The architecture of the small-residual-convolutional-network (SResCNN) model used for measuring PD-L1 expression non-invasively is presented in online supplemental figure S2. For each patient, only the primary tumor was analyzed. A square or an irregular-shaped box, which was close to the boundary of the tumor, was delineated manually in the aligned PET and CT images of the SPH cohort using ITK-SNAP^23^ software by experienced nuclear medicine radiologist (LJ). After dilation of the smallest square mask (SSM) including the selected region with a square of size 20 mm and resized to the size of 64×64 pixels using cubic spline interpolation, the PET region of interest (ROI) and CT ROI with the entire tumor and its peripheral region included were automatically generated at the same size (online supplemental figure S3). To reduce the effect of the difference between the central slice and peripheral slices, the area of each SSM within each patient was calculated, and only the SSMs with area larger than 30% of the maximum value were used to generate valid ROIs, which further constructed a three-channel hyper-images together with their fusion images (alpha-blending fusion,^24^ α=1, online supplemental figure S4). During the training of the model, 14,011 training hyper-images (6722 were PD-L1 positive and 7289 were PD-L1 negative) and 5291 validation hyper-images (2513 were PD-L1 positive and 2778 were PD-L1 negative) were used as the input images, the PD-L1 expression status (positive=1 or negative=0) was used as the label. After training, a DLS representing the PD-L1 positivity status was generated after a sequential activation of convolution and pooling layers. To develop a robust measurement, the average DLSs of all valid slices including tumor tissue fed into the SResCNN model with equal weight were regarded as the final PD-L1 positive probability of the tumor. Details of the building, training, optimization, and application methods were provided in online supplemental methods S3. The implementation of this model used the Keras toolkit and Python 3.5. The same pipeline (available at https://doi.org/10.5281/zenodo.4731166) was performed by an experienced radiologist (YS) on the three MCC cohorts and external VA cohort to obtain the DLS based on the guideline. Given there were minor differences between the different radiologists in selecting the ROIs, ROIs within the SPH validation cohort were also selected by YS again to validate the reproducibility of DLS. Regarding the importance of the hyper-image constructed with different modalities, similar SResCNN models using only PET or CT images were also trained.

### Visualization of the SResCNN model

To further understand the measurement processing and explore the biological underpinnings of the deep learning features, intermediate activation layers were first visualized to assess how the network carries the information from input to output.^25^ Additionally, the Gradient-weighted Class Activation Mapping (Grad-CAM) was used to understand the importance of each neuron for a decision of PD-L1 positive or negative and produce a coarse localization map highlighting the important regions in the image for predicting the target concept (PD-L1 positive or PD-L1 negative) by using the gradient information of target concept flowing into the last convolutional layer of the SResCNN model. And the reconstructed maps were named as positive and negative filter later, which were also used to evaluate the class discrimination.^26^ Besides, unsupervised hierarchical clustering was performed on the deeply learned features (ie, the output of global average pooling, N=256) to create a heatmap to show their distinguishable expression pattern among different patients. The clusters formed were based purely on the similarities and dissimilarities among the patients by the expressions of the deeply learned features.

### Statistical analysis

The Wilcoxon signed-rank test and Fisher’s exact test were used to test the differences for continuous variables and categorical variables, respectively. The area under the receiver operating characteristics curve (AUC), accuracy (ACC), sensitivity (SEN), specificity (SPEC), and the 95% CI by the DeLong method^27^ were used to assess the ability of DLS in discriminating between positive and negative PD-L1 expression. The cutoff was established using the maximum Youden index (ie, Specificity +Sensitivity-1) in the SPH training cohort. To compare the prognostic value of DLS with that of IHC-based PD-L1 status, the difference between HRs for the DLS and PD-L1 status computed by Cox regression model was calculated and evaluated with bootstrapped 95% CI.^28^ The inter-rater agreement of DLS estimations was calculated by intraclass correlation coefficient (ICC) between two radiologists. The bootstrapped mean value and SEs of the DLSs in different cohorts were also assessed for the similarity.

The correlation between DLS and different metadata (including age, body mass index, sex, stage, smoking status, Eastern Cooperative Oncology Group (ECOG) Performance Status, and SUVmax) and molecular features (including histology, PET/CT image-based necrosis, and PD-L1 TPS) were analyzed by Spearman’s rank correlation or point-biserial correlation. The details of necrosis quantification are shown in online supplemental methods S4. Comparison of the magnitude of two correlations was performed with a software package named cocor.^29^ Given prior research has suggested that PD-L1 expression is negatively correlated with EGFR mutation status,^30^ we contend that non-invasive methods of measuring PD-L1 and/or EGFR status would have clinical translational implications. Therefore, we also investigated whether the DLS was correlated with EGFR mutation status using point-biserial correlation and whether the DLS was affected by EGFR mutation status by comparing the performance in the subgroups divided by EGFR mutation status.

In the ICI-treated cohorts, the patients were clustered into high DLS and low DLS groups with the obtained cutoff, and survival analyses were performed using Kaplan-Meier method and Cox proportional hazards model. Using the MCC ICI-treated retrospective cohort, multivariable models, including the risk factors selected in univariate analysis according to the significance, were developed for the prediction of DCB, PFS, and OS, which were tested using the MCC ICI-treated prospective cohort as well as external VA ICI-treated retrospective cohort and were evaluated with C-indices. Z test was applied to compare the differences between different models. To rigorously assess the quality of the study design, the radiomic quality score was calculated^31^ (online supplemental methods S5 and table S2). Two-sided p values of less than 0.05 were regarded as significant, 10,000 replications were performed in bootstrap analyses, and all statistical analyses were conducted with IBM SPSS Statistics 25 (Armonk, New York, USA), R 3.6.3 (R Foundation for Statistical Computing, Vienna, Austria), and MATLAB R2019a (Natick, Massachusetts).

## Results

### Patients characteristics

The clinical characteristics of the patients used to train and test the non-invasive measurement of the PD-L1 status are presented in table 1 (online supplemental table S3 for external VA patients). The SPH training, SPH validation, and external MCC PD-L1 test cohorts used to train, validate, and test the SResCNN model had a prevalence of PD-L1 positivity by IHC of 29.93%, 30.17%, and 56.47%, respectively. The external VA patients had a significant higher PD-L1 positivity of 82.76% (within the 29 patients who had IHC PD-L1 expression).

**Table 1:** Demographic and clinical characteristics of patients used to measure PD-L1 status

Charac-teristic	SPH training cohort (N=284)				SPH validation cohort (N=116)				MCC PD-L1 test cohort (N=85)		
	PD-L1+	PD-L1−		P values	PD-L1+	PD-L1−		P values	PD-L1+	PD-L1−	P values
Age (years)				0.41				0.46			0.40
Mean±SD	62.71±8.78	63.51±8.56			63.6±9.28	62.73±8.84			68.21±9.18	64.59±14.61	
Sex, No (%)				0.035*				0.062			0.14
Male	58 (68.24)	108 (54.27)			26 (74.29)	44 (54.32)			25 (52.08)	26 (70.27)	
Female	27 (31.76)	91 (45.73)			9 (25.71)	37 (45.68)			23 (47.92)	11 (29.73)	
TNM stage				0.12				0.42			0.23
I	43 (50.59)	122 (61.31)			17 (48.57)	55 (67.9)			0 (0)	2 (5.41)	
II	22 (26.19)	34 (17.08)			9 (25.71)	14 (17.28)			2 (4.17)	0 (0)	
III	11 (13.10)	31 (15.58)			9 (25.71)	12 (14.81)			2 (4.17)	1 (2.70)	
IV	9 (10.71)	12 (6.03)			0 (0)	0 (0)			42 (87.50)	33 (89.19)	
Histology (baseline), No (%)				<0.001*				0.006*			0.83
ADC	48 (56.47)	156 (78.39)			19 (54.29)		65 (80.25)		26 (54.17)	21 (56.76)	
SCC	37 (43.53)	43 (21.61)			16 (45.71)		16 (19.75)		22 (45.83)	16 (43.24)	
EGFR, No (%)				0.020*				0.28			0.076
Mutation	24 (28.57)	87 (43.72)			9 (25.71)		32 (39.51)		2 (4.17)	5 (13.51)	
Wild	55 (65.48)	101 (50.75)			23 (65.71)		46 (56.79)		40 (83.33)	25 (67.57)	
ALK, No (%)				0.51				0.20			1.00
Mutation	1 (1.19)	1 (0.50)			2 (5.71)		1 (1.23)		1 (2.08)	0	
Wild	78 (92.86)	187 (93.97)			30 (85.71)		77 (95.06)		39 (8125)	29 (78.38)	
ROS1, No (%)				1.00				NaN			NaN
Mutation	0	2 (1.01)			0		0		0	0	
Wild	77 (91.67)	179 (89.95)			31 (88.57)		75 (92.59)		26 (54.17)	17 (45.95)	
Smoking status, No (%)				<0.001*				0.025*			0.83
Never	32 (37.65)	118 (59.3)			11 (31.43)		45 (55.56)		17 (35.42)	14 (37.84)	
Former	53 (62.35)	81 (40.7)			24 (68.57)		36 (44.44)		31 (64.58)	23 (62.16)	
SUVmax				<0.001*				0.003*			0.014*
Mean±SD	12.32±5.99		8.42±4.83		12.14±5.88		8.61±5.49		13.74±7.83	10.60±9.92	
Deeply learned score (DLS)				<0.001*				<0.001*			<0.001^*^
Median (IQR)	0.70 (0.60–0.78)		0.43 (0.30–0.55)		0.63 (0.55–0.71)		0.41 (0.270.52)		0.58 (0.47–0.62)	0.39 (0.26–0.43)	
PD-L1 positivity by IHC											
No (%)	85 (29.93)		199 (70.07)		35 (30.17)		81 (69.83)		48 (56.47)	37 (43.53)	

*Mean p value <0.05. The comparison of age and SUVmax between two groups was performed with Wikcoxon sign rank test, and the rest variables were compared with Fisher’s exact test. The demographic and clinical characteristics of external VA patients are provided in online supplemental table S2.

ADC, adenocarcinoma; ALK, anaplastic lymphoma kinase; EGFR, epidermal growth factor receptor; IHC, immunohistochemistry; MCC, H Lee Moffitt Cancer Center and Research Institute; NaN, not available; PD-L1, programmed death-ligand 1; ROS1, c-ros oncogene 1, receptor tyrosine kinase; SCC, squamous cell carcinoma; SPH, Shanghai Pulmonary Hospital; SUV, standardized uptake value; TNM, tumor, node, metastases; VA, James A Haley Veterans’ Administration.

The clinical characteristics of the patients used to test the clinical utility of DLS are presented in table 2. The retrospective MCC ICI-treated cohort included 128 patients with a median PFS and OS of 7.43 and 21.77 months, respectively, and 53.91% of the patients had DCB. The prospective MCC ICI-treated patients included 49 patients with a DCB rate of 65.31%, median PFS and OS of 10.50 and 17.00 months, respectively. For the external VA patients with a median PFS and OS of 8.13 and 13.10 months, 68.57% of the patients showed PD-L1 positive, and 54.29% of patients obtained DCB.

**Table 2:** Demographic and clinical characteristics for ICI-treated patients

Characteristic	Retrospective MCC ICI-treated patients (N=128)				Prospective MCC ICI-treated patients (N=49)			
	All	Deeply learned score		P values	All	Deeply learned score		P values
		High (N=43)	Low (N=85)			High (N=31)	Low (N=18)	
Age (years)				0.099		0.20		
Mean±SD	65.48±13.24	67.35±13.71	64.54±12.80		66.8±10.04	64.77±8.87	70.28±10.70	
BMI				0.43			0.48	
Mean±SD	26.14±5.08	25.60±4.79	26.42±5.17		26.06±5.02	26.05±5.46	26.08±4.00	
Sex, No (%)				0.38			0.56	
Male	81 (63.28)	24 (55.81)	57 (67.06)		25 (51.02)	17 (54.84)	8 (44.44)	
Female	47 (36.72)	19 (44.19)	28 (32.94)		24 (48.98)	14 (43.75)	10 (55.56)	
TNM stage				0.63			0.39	
III	25 (19.53)	11 (25.58)	14 (16.47)		6 (12.24)	5 (16.13)	1 (5.56)	
IV	103 (80.47)	32 (74.42)	71 (83.53)		43 (87.76)	26 (83.87)	17 (94.44)	
Histology (baseline), No (%)			0.096				0.23	
ADC	80 (62.50)	23 (53.49)	57 (67.06)		28 (57.14)	20 (64.52)	8 (44.44)	
SCC	48 (347.50)	20 (46.51)	28 (32.94)		21 (42.86)	11 (35.48)	10 (55.56)	
EGFR, No (%)				1.00				1.00
Mutation	8 (6.25)	2 (4.65)	6 (7.06)		2 (4.08)	2 (6.45)	0	
Wild	85 (66.41)	28 (65.12)	57 (67.06)		37 (75.51)	23 (74.19)	14 (77.78)	
ALK, No (%)				1.00				NaN
Mutation	2 (1.56)	0	2 (2.35)		0	0	0	
Wild	89 (69.53)	27 (62.79)	62 (72.94)		39 (79.59)	24 (77.42)	15 (83.33)	
ROS1, No (%)				NaN				NaN
Mutation	0	0	0		0	0	0	
Wild	35 (27.34)	7 (16.28)	28 (32.94)		33 (67.35)	20 (64.52)	13 (72.22)	
Smoke, No (%)			0.78				0.74	
Never	49 (38.28)	18 (41.86)	31 (36.47)		14 (28.57)	10 (32.26)	4 (22.22)	
Former	79 (61.72)	25 (58.14)	54 (63.53)		35 (71.43)	21 (67.74)	14 (77.78)	
ECOG scale, No (%)				0.43			0.49	
0	29 (22.66)	7 (16.28)	22 (25.88)		10 (16.33)	5 (16.13)	5 (27.78)	
1	91 (71.09)	34 (79.07)	57 (67.06)		38 (81.63)	25 (80.65)	13 (42.22)	
≥2	8 (6.25)	2 (4.65)	6 (7.06)		1 (2.04)	1 (323)	0 (0)	
SUVmax			0.014*				0.15	
Mean±SD	11.82±6.98	13.44±5.83	11.00±7.32		14.59±9.53	14.49±6.37	14.77±13.13	
Clinical benefit, No (%)			<0.001*				0.005*	
DCB	69 (53.91)	34 (79.07)	35 (41.18)		32 (65.31)	25 (80.65)	7 (38.89)	
NDB	59 (46.09)	9 (20.93)	50 (58.82)		17 (34.69)	6 (19.35)	11 (61.11)	
Progression-free survival			<0.001*				0.015*	
Median (95% CI)	7.43 (6.39 to 8.47)	15.80 (9.49 to 22.11)	5.50 (2.87 to 8.13)		10.50 (6.36 to 14.64)	14.33 (8.57 to 20.10)	5.00 (2.84 to 7.16)	
Overall survival			0.021*				<0.001^*^	
Median (95% CI)	21.77 (13.50 to 30.03)	27.60 (NR)	19.77 (13.82 to 25.72)		17.00 (NR)	NR	11.23 (6.69 to 15.78)	
Deep learning score			<0.001*				<0.001*	
Median (IQR)	0.48 (0.01–0.93)	0.63 (0.60–071)	0.42 (0.32–0.48)		0.55 (0.14–0.86)	0.59 (0.56–064)	0.34 (0.31–0.45)	

*p value<0.05. The comparison of age, BMI, and SUVmax between two groups was performed with Wilcoxon sign rank test, PFS and OS were compared with log-rank test, and the rest variables were compared with Fisher’s exact test.

ADC, adenocarcinoma; ALK, anaplastic lymphoma kinase; BMI, body mass index; DCB, durable clinical benefit; ECOG, Eastern Cooperative Oncology Group Performance Status; EGFR, epidermal growth factor receptor; ICI, immune checkpoint inhibitor; MCC, H Lee Moffitt Cancer Center and Research Institute; NDB, non-durable benefit; NR, not reached; SCC, squamous cell carcinoma; TNM, tumor, node, metastases.

### Association between DLS, PD-L1, and metadata

**Figure 2:**
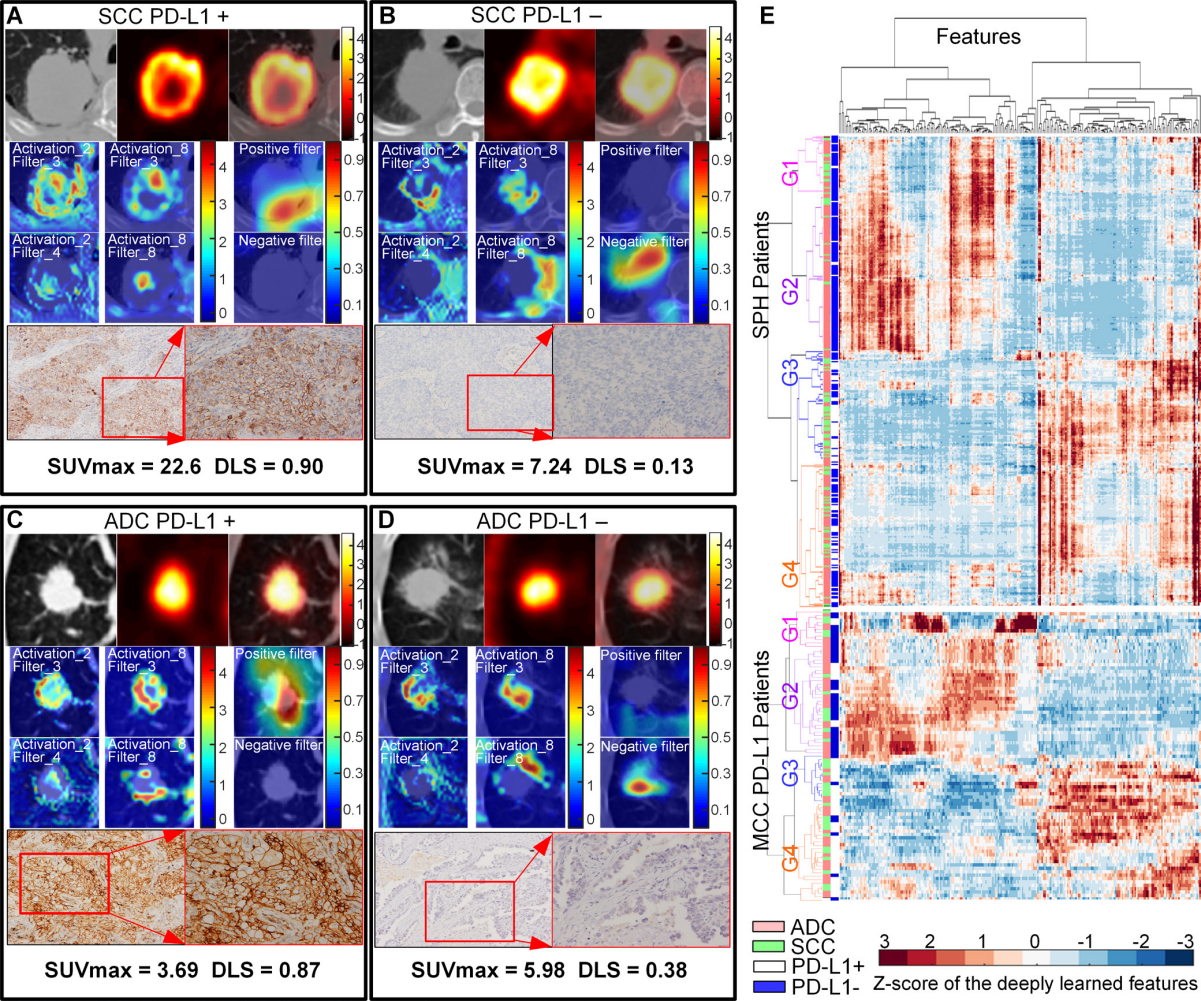
NSCLC histology subtypes and PD-L1 expression. Squamous cell carcinoma (SCC) patients with positive PD-L1 expression (A) and negative PD-L1 expression (B). Adenocarcinoma (ADC) patients with positive PD-L1 expression (C) and negative PD-L1 expression (D), respectively. For (A)–(D), the first line is the CT, PET, and fusion images, the first and second columns of the second and third line show the response of the fourth ResBlock, which shows the self-learned important areas in expressing PD-L1 status (peritumoral and necrosis regions), the third column of the second and third line shows the response of the negative filter and the positive filter in the PD-L1 positive–negative tumors (the CT images were overlapped to reveal the location of the response), the last line shows the pathological examination of the resected mass demonstrating PD-L1 expression (left, ×100; right, ×200). (E) The heatmap generated with unsupervised hierarchical clustering of all the SPH patients and MCC PD-L1 patients on the horizontal axis and deeply learned features expression (ie, the output of the last activation filters, N=256) on the vertical axis. There were four distinct subgroups obtained. Groups G1 and G2 (including more PD-L1− patients) had similar feature expression, which is opposite to the feature expression of G3 and G4 (including more PD-L1+ patients). Furthermore, some features of G1 and G2 (or G3 and G4) are different. G1 and G3 had more patients with SCC, while G2 and G4 had more patients with ADC. The χ^2^ test showed the significant association of the four kinds of deep learning expression patterns with PD-L1 expression (SPH patients: p*<*0.001, MCC patients: p*<*0.001) and different histology (SPH patients: p*<*0.001, MCC patients: p*=*0.061). The similar patterns of the external MCC PD-L1 cohorts further showed the stability of the deep learning features. ADC, adenocarcinoma; PD-L1, programmed death-ligand 1; SUV, standardized uptake value; DLS, deeply learned score; MCC, H Lee Moffitt Cancer Center and Research Institute; NSCLC, non-small cell lung cancer; SCC, squamous cell carcinoma; SPH, Shanghai Pulmonary Hospital

The DLS exhibited statistically significant differences between the PD-L1-positive and PD-L1-negative tumors in all three cohorts (p*<*0.001), and four examples are shown in figure 2 (adapted from Mu *et al* 17). The DLS was also positively correlated with the original PD-L1 TPS in both SPH (Spearman’s rho=0.60, p<0.001) and MCC PD-L1 test (Spearman’s rho=0.59, p<0.001) cohorts, which was significantly higher compared with the correlation between the SUVmax and the TPS with rho of 0.30 (p<0.001) and 0.29 (p=0.009), respectively. Using analysis of variance, the DLS was significantly different between groups with PD-L1 TPS <1%, 1%–49%, and ≥50% (SPH cohort: p<0.001; MCC PD-L1 test cohort: p<0.001). The least squares difference (LSD) post hoc analysis showed significantly higher values of DLS in patients with PD-L1 TPS ≥50% than TPS 1%–49% group (LSD: SPH cohort: p<0.017; MCC PD-L1 test cohort: p≤0.027) and TPS <1% group (LSD: SPH cohort: p<0.001; MCC PD-L1 test cohort: p<0.001) (details are shown in online supplemental figure S5). As such, the increased PD-L1 TPS scores correlated to the DLS.

Additionally, the DLS was positively correlated with SUVmax (Spearman’s rho=0.43, p<0.001), squamous cell carcinoma (SCC) (point-biserial *rho_pb_* =0.27, p<0.001), male sex (point-biserial *rho_pb_* =0.19, p<0.001), smoking status (point-biserial *rho_pb_* =0.20, p<0.001), and negatively correlated with *EGFR* status (point-biserial *rho_pb_* =−0.20, p<0.001) for the whole SPH cohort. In the MCC PD-L1 test cohort, the only positive significant correlation was with SUVmax (Spearman’s rho=0.34, p<0.001) and negative with *EGFR* status (point-biserial *rho_pb_* =−0.25, p=0.035). Further, multivariable linear regression (adjusted r^2^=0.15, F=15.31, p<0.001) showed that only SUVmax (coefficient=0.32, p=0.005) was independently associated with the DLS. Only 15% of DLS variability could be explained by the SUVmax, indicating that DLS originated mainly from other image information.

Through the visualization of the SResCNN model, the necrotic region was identified as self-learned important area for classifying PD-L1 status (figure 2A). Quantitatively, for all the SPH patients with necrotic regions, a significant correlation was observed between the necrosis-to-global volume ratio of the PET images and DLS with Spearman’s rho of 0.50 (p<0.001) (online supplemental figure S6). Further, univariable linear regression (adjusted r^2^=0.24, F=15.92, p<0.001) showed that the necrosis (coefficient=0.49, p<0.001) was independently associated with DLS and could explain 24% of DLS variability. Therefore, necrosis potentially played an important role in predicting PD-L1 status.

Finally, the DLS was not correlated with tumor volume, neither in the entire SPH cohort (rho=0.082, p=0.10) and nor in the MCC PD-L1 test cohort (rho=−0.066, p=0.55), which indicates the T stage has a limited effect on DLS in this study.

### Analytical validation of DLS in predicting PD-L1 status

**Figure 3:**
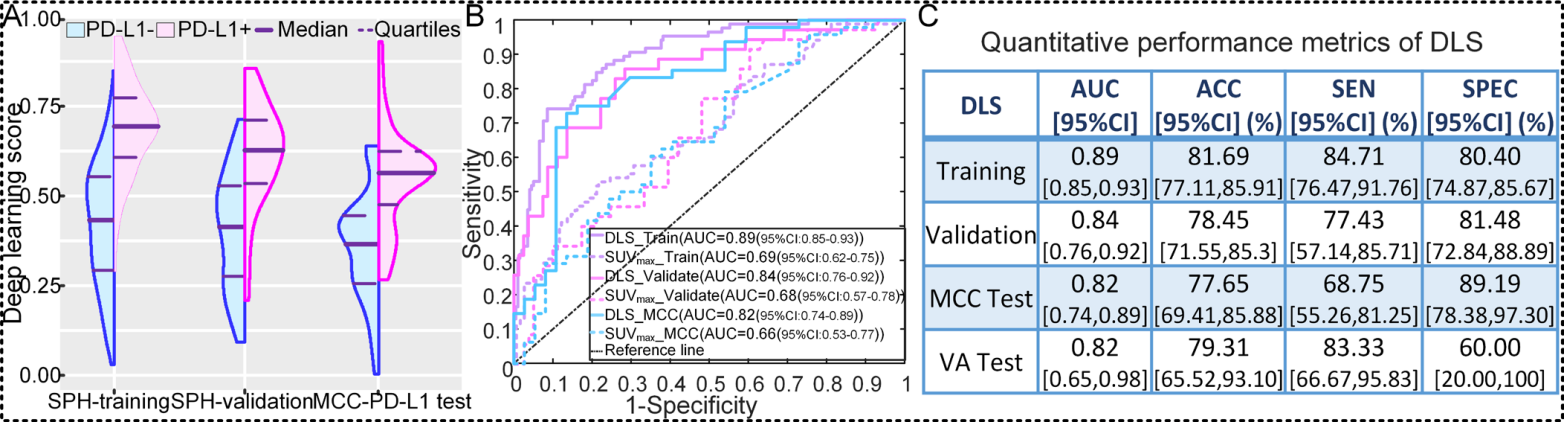
Performance of the DLS in predicting PD-L1 status. (A) The distribution of DLS between PD-L1-positive (+) and PD-L1-negative (−) groups in SPH training, SPH validation, and external MCC PD-L1 test cohorts. (B) The receiver operating characteristic curves of DLS and SUVmax in SPH training, SPH validation, and external MCC PD-L1 test cohorts. (C) The quantitative performance metrics in SPH training, SPH validation, external MCC PD-L1 test, and external VA test cohorts. ACC, accuracy; AUC, area under receiver operating characteristics curve; DLS, deeply learned score; PD-L1, programmed death-ligand 1; SUV, standardized uptake value; MCC, H Lee Moffitt Cancer Center and Research Institute; SEN, sensitivity; SPEC, specificity; SPH, Shanghai Pulmonary Hospital; VA, James A Haley Veterans’ Administration.

To discriminate PD-L1-positive from PD-L1-negative expression, the DLS yielded AUCs of 0.89 (95% CI: 0.84 to 0.94; p*<*0.001), 0.84 (95% CI: 0.76 to 0.92; p*<*0.001), and 0.82 (95% CI: 0.74 to 0.89; p*<*0.001), accuracies of 81.69% (95% CI: 77.11% to 85.91%), 78.45% (95% CI: 71.55% to 85.30%), and 77.65% (95% CI: 69.41% to 85.88%), sensitivities of 84.71% (95% CI: 76.47% to 91.76%), 77.43% (95% CI: 57.14% to 85.71%), and 68.75% (95% CI: 55.26% to 81.25%), specificities of 80.40% (95% CI: 74.87% to 85.67%), 81.48% (95% CI: 72.84% to 88.89%), and 89.19% (95% CI: 78.38% to 97.30%) in the SPH training, SPH validation, and MCC PD-L1 test cohorts, respectively, with a cutoff value of 0.55 (figure 3 and online supplemental table S4). For the external VA PD-L1 patients, the DLS generated an AUC of 0.82 (95% CI: 0.65 to 0.98; p=0.028), accuracy of 79.31% (95% CI: 62.07% to 93.10%), sensitivity of 83.33% (93% CI: 66.67% to 95.83%), and specificity of 60.00% (95% CI: 20.00% to 100%) (figure 3C and online supplemental table S4).

As another meaningful quantitative index associated with PD-L1 expression validated in other studies,^32^ SUV_max_ showed poorer performance to discriminate between PD-L1-positive and PD-L1-negative expression with AUCs of 0.69 (95% CI: 0.62 to 0.75; p<0.001), 0.68 (95%CI: 0.57 to 0.78; p<0.001), 0.66 (95%CI: 0.53 to 0.77; p=*0*.014), and 0.56 (95%CI: 0.28 to 0.84; p=0.69) in the SPH training, SPH validation, external MCC PD-L1 test and VA PD-L1 test cohorts, respectively.

Since histology was found to be significantly associated with PD-L1 status (p<0.01), a stratified analysis was conducted to assess the SResCNN model in predicting PD-L1 status by histology (online supplemental table S4). The results from these analyses indicated this model also performed well in both adenocarcinoma (ADC) and SCC lung cancers. Additionally, although there was significant negative association between DLS and *EGFR* mutation status, the DLS yielded high AUCs of 0.90 (95% CI: 0.84 to 0.98), 0.87 (95% CI: 0.73 to 1.00), and 0.80 (95% CI: 0.42 to 1.00) in patients with an *EGFR* mutation and also high, but not statistically significant different AUCs of 0.88 (95% CI: 0.83 to 0.94, DeLong test p=0.71), 0.85 (95% CI: 0.75 to 0.95, DeLong test p=0.61), and 0.82 (95% CI: 0.70 to 0.93, DeLong test p=0.93) in patients with wild type of *EGFR* in the SPH training, SPH validation, and MCC PD-L1 test cohorts, respectively.

Performance of the PD-L1 DLS was further investigated by comparing its prognostic to that of PD-L1 IHC. Among 85 patients in the MCC PD-L1 test cohort and 29 patients in the external VA test cohort that have PD-L1 IHC data, the DLS achieved AUCs of 0.70 (95% CI: 0.60 to 0.82) and 0.68 (95% CI: 0.47 to 0.89) in DCB prediction for MCC and VA, respectively. These were nearly identical to DCB prediction by PD-L1 IHC, which exhibited AUCs of 0.67 (95% CI: 0.55 to 0.78, DeLong test p=0.45) and 0.69 (95% CI: 0.49 to 0.90, DeLong test p=0.94), respectively. Among patients in the MCC PD-L1 test cohort, the HRs for PFS and OS in the high DLS subgroup (DLS ≥0.55) were 0.39 (95% CI: 0.22 to 0.69, p=0.001) and 0.26 (95% CI: 0.11 to 0.62, p=0.002), which had small difference of 0.048 (95% CI: 0.001 to 0.18) and 0.038 (95% CI: 0.00 to 0.13) compared with that of PD-L1-positive group (HR for PFS: 0.42 (95% CI: 0.26 to 0.73, p=0.002); HR for OS: 0.30 (95% CI: 0.14 to 0.64, p=0.002)), respectively. Similar results were observed among the external VA test cohort where the difference between the HRs of high DLS (HR=0.35 (95% CI: 0.12 to 1.06, p=0.063)) and positive IHC-based PD-L1 status (HR=0.21 (95% CI: 0.060 to 0.77, p=0.018)) for PFS was as small as 0.14 (95% CI: 0 to 0.69). The HRs of high DLS for OS was 0.26 (95% CI: 0.079 to 0.87, p=0.029), which had also small difference of 0.056 (95% CI: 0 to 0.22) compared with that of positive IHC-based PD-L1 status with HR of 0.21 (95% CI: 0.057 to 0.76, p=0.018). Therefore, the ability of the DLS and IHC metrics was indistinguishable in their ability to predict PFS and OS in response to ICI.

Regarding the stability of the DLS, though accurate segmentations were not needed, radiologists had to delineate a rough ROI that contained the tumors and some surrounding tissue. To investigate the effect of the minor differences between the different radiologists in selecting the rough ROIs, the ROIs of the SPH validation patients (n=116 cases) were generated by two radiologists, and two DLSs were obtained accordingly. The ICC of these two DLSs was as high as 0.85 (95% CI: 0.80 to 0.90, p<0.001). Further, similar bootstrapped mean values of the DLSs were found across the five patient cohorts (online supplemental figure S7).

The resulting DLSs of PET-based SResCNN model and CT-based SResCNN model, respectively, achieved AUCs of 0.81 and 0.78 in the training cohort, 0.73 and 0.70 in the validation cohort, which were significantly worse (DeLong test p<0.001) than those generated using the hyper-images.

### Clinical prognostic validation of DLS in ICI treatment

**Figure 4:**
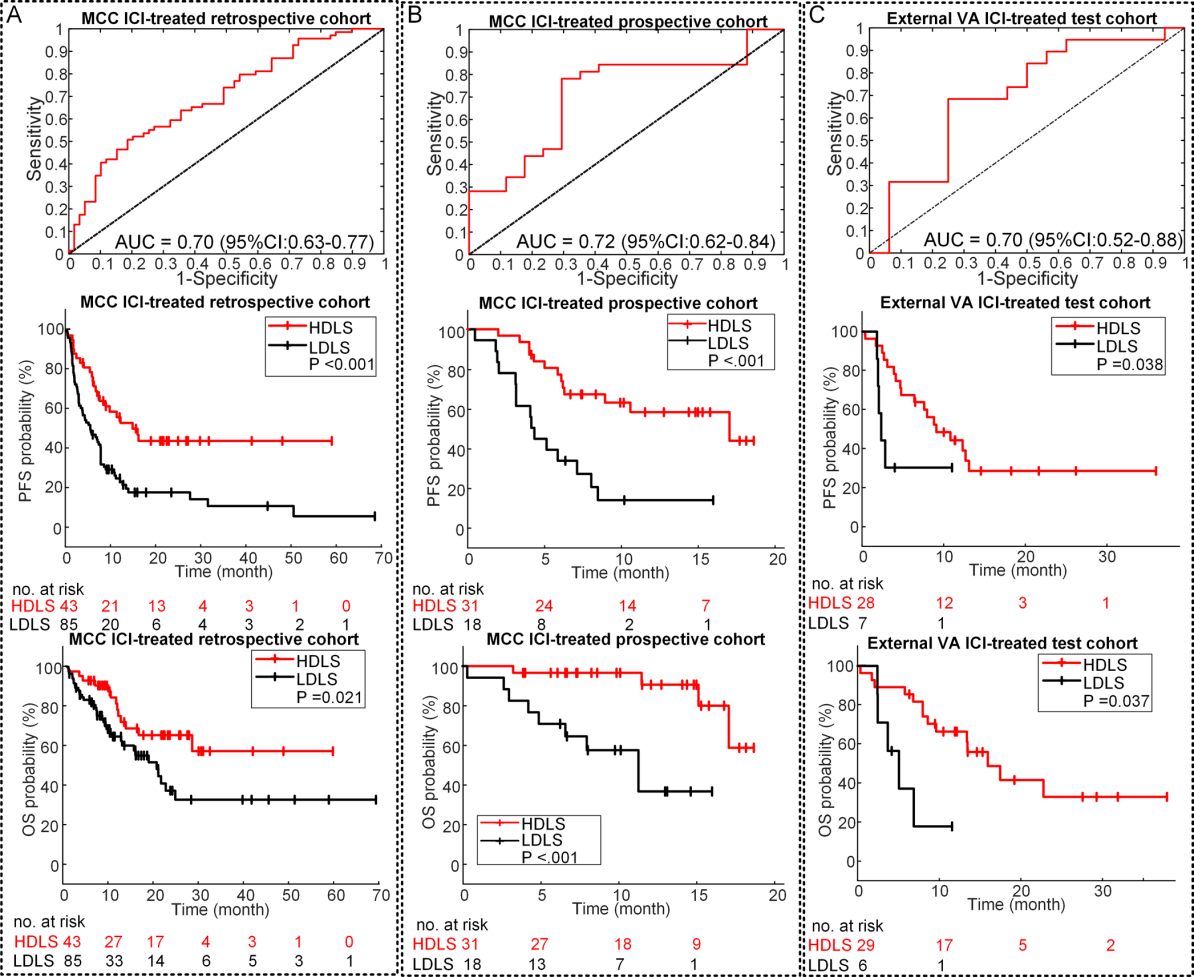
Performance of the DLS in prognosis prediction. (A) The ROC curve of DLS in DCB prediction, and the PFS and OS relative to the DLS (DLS cutoff: 0.55) in the retrospective MCC ICI-treated patients. (B) The ROC curve of DLS in DCB prediction, and the PFS and OS relative to the DLS (DLS cutoff: 0.55) in the prospective MCC ICI-treated patients. (C) The ROC curve of DLS in DCB prediction, and the PFS and OS relative to the DLS (DLS cutoff: 0.55) in the external VA test patients. P value was from log rank test. AUC, area under the receiver operating characteristic curve; DCB, durable clinical benefit; DLS, deeply learned score; HDLS, high DLS; ICI, immune checkpoint inhibitor; LDLS, low DLS; MCC, H Lee Moffitt Cancer Center and Research Institute; OS, overall survival; PFS, progression-freesurvival; ROC, receiver operating characteristic; VA, James A Haley Veterans’ Administration.

The DLS in the patients experiencing DCB was significantly higher compared with those who did not in both the MCC ICI-treated retrospective (0.54 vs 0.43, p<0.001) and prospective (0.57 vs 0.45, p=0.025) cohorts. The AUCs of the DLS to identify the DCB patients were 0.70 (95% CI: 0.63 to 0.77, p<0.001) and 0.72 (95% CI: 0.62 to 0.84, p=0.014) in the retrospective and prospective patients (figure 4A, B), respectively. Similar results were obtained in the external VA test cohort with an AUC of 0.70 (95% CI: 0.52 to 0.88, p=0.040) (figure 4C).

For the retrospective patients, the PFS and OS were significantly longer among patients with high DLS (≥0.55) versus patients with low DLS (PFS: HR=0.41 (95% CI: 0.25 to 0.67, p=0.001); OS: HR=0.48 (95% CI: 0.25 to 0.91, p=0.024); figure 4A). Among patients with high DLS, the median PFS and OS were 15.80 months and 27.60 months compared with 5.37 months and 19.77 months for patients with low DLS (PFS: p<0.001; OS: p=0.021). Similar results were also observed in the prospective patients with high to low DLS ratio-based HRs of 0.38 (95% CI: 0.18 to 0.85, p=0.019) and 0.13 (95% CI: 0.033 to 0.49, p=0.003) for PFS and OS, respectively (figure 4B). High DLS patients had a longer median PFS of 14.33 months compared with 5.00 months in the low DLS patients (p<0.001). Notably, a median time to an OS event was not reached in the high DLS group and was 11.23 months in the low DLS group (p<0.001). The external VA test patients further validate the prognostic value of DLS with HRs of 0.35 (95% CI: 0.12 to 0.99, p=0.047) and 0.23 (95% CI: 0.07 to 0.72, p=0.020) for PFS (9.30 vs 2.37 months, p=0.038) and OS (15.53 vs 4.93 months, p=0.007), respectively (figure 4C).

### Multivariable analysis for clinical outcomes prediction

**Figure 5:**
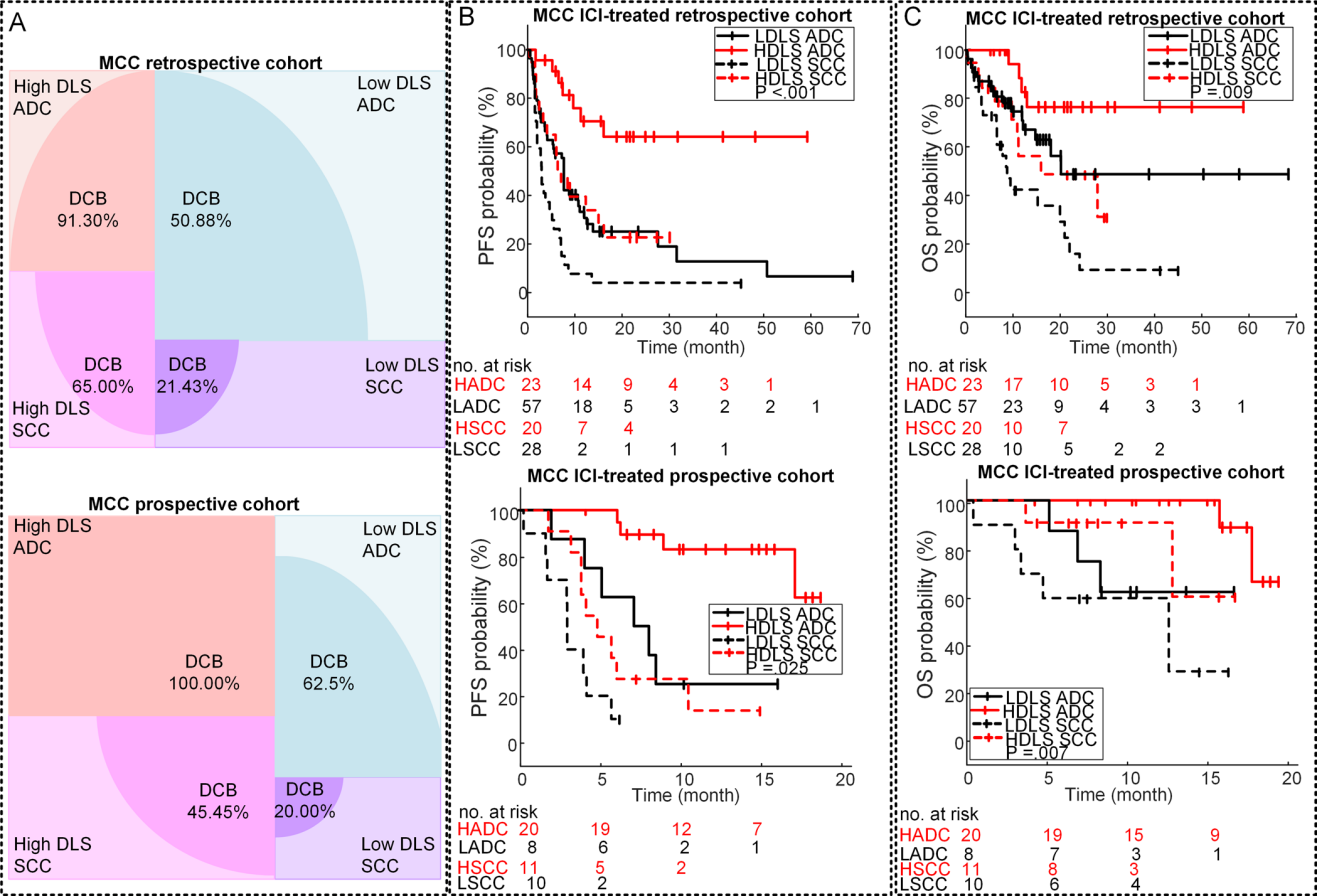
Stratification analysis of the performance of the DLS in prognosis prediction. (A) The DCB rates of the different subgroups of the MCC retrospective and prospective ICI-treated patients. (B) The PFS relative to the DLS and histology in the MCC retrospective and prospective ICI-treated patients. (C) The OS relative to the DLS and histology in the MCC retrospective and prospective ICI-treated patients. Note: HADC is short for HDLS ADC, meaning ADC patients with high DLS; LADC is short for LDLS ADC, meaning ADC patients with low DLS; HSCC is short for HDLS SCC, meaning SCC patients with high DLS; and LSCC is short for LDLS SCC, meaning SCC patients with low DLS, the high DLS versus low DLS defined by 0.55. P value was from log rank test. ADC, adenocarcinoma; DCB, durable clinical benefit; DLS, deeply learned score; ICI, immune checkpoint inhibitor; MCC, H Lee Moffitt Cancer Center and Research Institute; OS, overall survival; PFS, progression-freesurvival; SCC, squamouscell carcinoma.

Univariable logistic and Cox regression analyses of the clinical characteristics (online supplemental tables S5–S8) and gene mutation showed that none of three gene mutations were associated with clinical outcome and that patients with lower ECOG status and ADC showed significantly longer OS and PFS. Stratified analyses by histology and ECOG performance status were thus performed to investigate the ability of DLS to predict outcomes in these subgroups. Among patients with ADC, the DCB rates were 91.3% and 100% in patients with high DLS versus 50.88% and 62.5% in patients with low DLS in both retrospective and prospective cohorts (p<0.001), respectively (figure 5). Among SCC patients, though the DCB rates were lower compared with ADC, the patients with higher DLS still had a significantly higher DCB rates in both retrospective and prospective cohorts. Congruously, the PFS and OS of high DLS group were also longer than the low DLS group in both ADC and SCC subgroups (online supplemental table S9). The results of the stratified analysis based on ECOG status (online supplemental table S10) also showed that low DLS was still associated with poor outcomes among patients with high ECOG status (≥1). The above results demonstrated the added value of DLS to the clinical prognostic markers in more accurate quantitative prognosis prediction.

Multivariable logistic regression and Cox proportional hazards regression analyses were conducted to adjust for potential confounding variables. Models including DLS, histology, and ECOG status were developed using the MCC ICI-treated retrospective cohort, which demonstrated high performance statistics (online supplemental table S11 and figure S8) with C-indices of 0.87 (95% CI: 0.83 to 0.92, p<0.001) and 0.82 (95% CI: 0.72 to 0.92, p<0.001) in DCB prediction, 0.73 (95% CI: 0.68 to 0.78, p<0.001) and 0.74 (95% CI: 0.67 to 0.87, p<0.001) in PFS prediction, 0.77 (95% CI: 0.71 to 0.84, p<0.001) and 0.70 (95% CI: 0.50 to 0.87, p<0.001) in OS prediction for the MCC ICI-treated retrospective cohort and independent MCC ICI-treated prospective cohort, respectively, showing better performance than clinical characteristics only models (including ECOG and histology) (p≤0.05, online supplemental table S12). These models also demonstrated high performance statistics with C-indices of 0.81 (95% CI: 0.70 to 0.93, p<0.001), 0.70 (95% CI: 0.59 to 0.80, p<0.001), and 0.70 (95% CI: 0.59 to 0.81, p<0.001) in DCB, PFS, and OS prediction, respectively, in the independent external VA patients. The calibration curves of the different models on MCC ICI-treated retrospective cohort, independent MCC ICI-treated prospective cohort, and the external VA cohort provided in online supplemental figure S9 also showed good agreements between the predictions and actual observation.

## Discussion

PD-L1 expression status based on IHC is currently used as a clinical decision-making tool to support the use of checkpoint inhibitors in patients with NSCLC. Because this relies on invasive biopsies, an alternative non-invasive method to predict PD-L1 status would be useful. In this study, we developed a deep learning model using standard-of-care PET/CT images to measure PD-L1 status non-invasively and showed that the DLS could discriminate between positive and negative expression with an AUC of 0.89 in the SPH training cohort, 0.84 in the SPH validation cohort, and 0.82 in the two independent MCC PD-L1 and VA PD-L1 test cohorts. When the DLS was combined with clinical covariates and tested for clinical utility by identifying patients most likely to benefit to immunotherapy, we found high C-indices of 0.81–0.87 for predicting DCB, but somewhat attenuated C-indices of 0.70–0.77 for the DLS to predict PFS and OS in the MCC ICI-treated retrospective and two independent MCC ICI-treated prospective and VA ICI-treated cohorts.

Others have investigated the utility of radiomics as a non-invasive approach to predict PD-L1 expression status.^33 34^ The current work is a significant advance over these prior studies, which were limited to a single institution, did not validate against independent cohorts, and used cohorts with many early-stage cancers included that are not candidates for ICI therapy. Further, our statistical power outperformed both of these prior studies, which generated AUCs of 0.86 and 0.73, respectively. As an alternative to PD-L1 as a companion biomarker, it should be recognized that tumor mutational burden (TMB), defined as the number of mutations per DNA megabase, is also promising biomarker for predicting immunotherapy responses in patients with advanced stage lung cancer.^35^ He *et al* 36 developed and tested a non-invasive CT-based TMB predictor with 327 patients, which yielded high prognostic value in PFS and OS prediction of immunotherapy in patients with advanced NSCLC. Despite these findings, TMB is not yet a clinically approved diagnostic biomarker attributed in part to the lack of harmonization in panel-based TMB quantification, adequate methods to convert TMB estimates across different panels, and robust predictive cutoff points.^37^

We and others have developed radiomic models to predict lung cancer immunotherapy treatment response regardless of PD-L1 status.^38–41^ Some of these have higher accuracies than do the current model in predicting DCB, PFS, or OS following ICI therapy. However, it is important to note that there is a distinction between the development and application of a completely new companion biomarker such as these, compared with one that provides an alternative assay to assess a currently approved companion biomarker as in the current study. We contend that a radiomic biomarker that predicts PD-L1 status will be more readily accepted into clinical practice compared with a radiomic biomarker that bypasses this known pathway. The current work is the first to develop a PD-L1 radiomic signature and then to use this for response prediction. Additionally, these prior studies were mostly limited to CT and required explicit tumor segmentation, which can render the results to be operator dependent. By contrast, our study utilized deep learning, which did not require accurate tumor segmentation or hard-coded feature extraction and was conducted using rigorous training and validation in multiple cohorts from three institutions. The current study is the single largest multi-institutional radiomic study population of patients with NSCLC to date treated with immunotherapy to predict PD-L1 status and subsequent treatment response using ^18^F-FDG PET/CT.

In radiomics, it is critical to relate the findings to an underlying biology. One of the high-response areas of the middle layer of the SResCNN model recognized the necrotic region (activation_8_filter_8 in figure 2A, B) through the visualization,^26^ suggesting that some final discriminant deeply learned features originate from necrotic regions, which is consistent with the correlation between necrosis and DLS and Jreige’s results.^11^ This could be explained with the presence of hypoxia, which can lead to necrotic cell death^42^ and upregulate PD-L1 via hypoxia-inducible factor-1α.^43^ Additionally, peritumoral regions were also highlighted as informative (activation_8_filter_8 and positive/negative filter in figure 2C, D), which is supported by prior work that higher levels of PD-L1+ staining in cells of peritumoral areas.^44^ These findings revealed an advantage of deeply learned models, which can agnostically capture features from the tumor and peritumoral microenvironments. One possible reason for the significant better predictive ability of the hyper-image compared with PET or CT alone may be these two important regions could be better and easier localized by utilizing both metabolic and anatomical information as reflected by PET and CT images, respectively.

We do acknowledge some limitations of this study. First, the PD-L1 prediction training data were limited to a single institution and *EGFR* mutations were highly prevalent in the Asian patient population at 40% compared with only 7% in whites. This concern is somewhat mitigated by the observation of no significant association between ethnicities and PD-L1 status,^45^ and the insignificant different AUCs between mutated *EGFR* subgroups and wild-type *EGFR* subgroups. Second, compared with other PD-L1 level detection methods, such as ELISA,^46^ immunofluorescence,^47^ and flow cytometry,^48^ only IHC was used in this study to detect PD-L1 expression levels based on the recommendation in the NCCN Clinical Practice Guidelines,^3^ its ease of use, strong repeatability, and high accuracy.^49 50^ Comparison among different detection methods should be considered in future research although these other methods are also dependent on biopsy. Third, the patient cohorts were heterogeneous in terms of PET/CT image acquisition. However, this can be viewed as a strength, as this heterogeneity decreases the possibility of overfitting to a specific subset of tumors or imaging parameters, and thus will result in a model that is more robust and transportable. Fourth, the stage distribution was different between the SPH and the MCC cohorts, as the MCC cohort contained more advanced stage patients. To investigate this, we measure the DLS among the subset of SPH patients with advanced stage and obtained high AUCs of 0.90 (95% CI: 0.85 to 0.97, p<0.001), suggesting that stage does not dramatically affect the final DLS prediction.

## Conclusion

In conclusion, an effective and stable deeply learned score to measure PD-L1 expression status non-invasively was identified and may serve as a prognostic biomarker to guide immunotherapy. Because images are routinely obtained and are not subject to sampling bias per se, we propose that the individualized risk assessment information provided by these analyses may be useful as a future clinical decision support tool pending in larger prospective trials.

## Data Availability

Data are available upon reasonable request. The PET/CT imaging data and clinical information are not publicly available for patient privacy purposes, but are available from the corresponding authors upon reasonable request (RJG and MBS). The remaining data are available within the Article, Supplementary Information or available from the authors upon request. The models and the code used to test and evaluate the model are available on Zenodo (https://doi.org/10.5281/zenodo.4731166).

## References

[1] Rittmeyer A , Barlesi F , Waterkamp D , et al . Atezolizumab versus docetaxel in patients with previously treated non-small-cell lung cancer (oak): a phase 3, open-label, multicentre randomised controlled trial. Lancet 2017;389:255–65. 10.1016/S0140-6736(16)32517-X 27979383 PMC6886121

[2] Gandhi L , Rodríguez-Abreu D , Gadgeel S , et al . Pembrolizumab plus chemotherapy in metastatic non-small-cell lung cancer. N Engl J Med 2018;378:2078–92. 10.1056/NEJMoa1801005 29658856

[3] National Comprehensive Cancer Network (NCCN) . NCCN Clinical Practice Guidelines in Oncology. Non-small Cell Lung Cancer version 4.2021, 2021.

[4] Topalian SL , Hodi FS , Brahmer JR , et al . Safety, activity, and immune correlates of anti-PD-1 antibody in cancer. N Engl J Med 2012;366:2443–54. 10.1056/NEJMoa1200690 22658127 PMC3544539

[5] Yu H , Boyle TA , Zhou C , et al . PD-L1 expression in lung cancer. J Thorac Oncol 2016;11:964–75. 10.1016/j.jtho.2016.04.014 27117833 PMC5353357

[6] Rossi S , Toschi L , Castello A , et al . Clinical characteristics of patient selection and imaging predictors of outcome in solid tumors treated with checkpoint-inhibitors. Eur J Nucl Med Mol Imaging 2017;44:2310–25. 10.1007/s00259-017-3802-5 28815334

[7] Liu Y , Kim J , Qu F , et al . Ct features associated with epidermal growth factor receptor mutation status in patients with lung adenocarcinoma. Radiology 2016;280:271–80. 10.1148/radiol.2016151455 26937803 PMC4934516

[8] Grossmann P , Stringfield O , El-Hachem N , et al . Defining the biological basis of radiomic phenotypes in lung cancer. Elife 2017;6. 10.7554/eLife.23421. [Epub ahead of print: 21 07 2017]. PMC5590809 28731408

[9] Herbst RS , Soria J-C , Kowanetz M , et al . Predictive correlates of response to the anti-PD-L1 antibody MPDL3280A in cancer patients. Nature 2014;515:563–7. 10.1038/nature14011 25428504 PMC4836193

[10] Chen DS , Mellman I . Elements of cancer immunity and the cancer-immune set point. Nature 2017;541:321–30. 10.1038/nature21349 28102259

[11] Jreige M , Letovanec I , Chaba K , et al . ^18^F-FDG PET metabolic-to-morphological volume ratio predicts PD-L1 tumour expression and response to PD-1 blockade in non-small-cell lung cancer. Eur J Nucl Med Mol Imaging 2019;46:1–10. 10.1007/s00259-019-04348-x 31214790

[12] Koh YW , Han J-H , Park SY , et al . Glut1 as a prognostic factor for classical Hodgkin's lymphoma: correlation with PD-L1 and PD-L2 expression. J Pathol Transl Med 2017;51:152–8. 10.4132/jptm.2016.11.03 28219001 PMC5357756

[13] Cui Y , Li Y , Li X . The impact of PD-L1 on glucose metabolism of lung adenocarcinoma cells. J Nucl Med 2018;59:1252–52.

[14] Damgaci S , Ibrahim-Hashim A , Enriquez-Navas PM , et al . Hypoxia and acidosis: immune suppressors and therapeutic targets. Immunology 2018;154:354–62. 10.1111/imm.12917 29485185 PMC6002221

[15] Lopci E , Toschi L , Grizzi F , et al . Correlation of metabolic information on FDG-PET with tissue expression of immune markers in patients with non-small cell lung cancer (NSCLC) who are candidates for upfront surgery. Eur J Nucl Med Mol Imaging 2016;43:1954–61. 10.1007/s00259-016-3425-2 27251642

[16] Wu X , Huang Y , Zhao Q , et al . PD-L1 expression correlation with metabolic parameters of FDG PET/CT and clinicopathological characteristics in non-small cell lung cancer. EJNMMI Res 2020;10:1–9. 10.1186/s13550-020-00639-9 32430866 PMC7237589

[17] Mu W , Jiang L , Zhang J , et al . Non-invasive decision support for NSCLC treatment using PET/CT radiomics. Nat Commun 2020;11:5228. 10.1038/s41467-020-19116-x 33067442 PMC7567795

[18] Health UDo, Services H . Software as a medical device (SAMD): clinical evaluation. Guidance for industry and Food and Drug Administration Staff, 2017.

[19] Rizvi NA , Hellmann MD , Snyder A , et al . Cancer immunology. mutational landscape determines sensitivity to PD-1 blockade in non-small cell lung cancer. Science 2015;348:124–8. 10.1126/science.aaa1348 25765070 PMC4993154

[20] Eisenhauer EA , Therasse P , Bogaerts J , et al . New response evaluation criteria in solid tumours: revised RECIST guideline (version 1.1). Eur J Cancer 2009;45:228–47. 10.1016/j.ejca.2008.10.026 19097774

[21] Rizvi H , Sanchez-Vega F , La K , et al . Molecular Determinants of Response to Anti-Programmed Cell Death (PD)-1 and Anti-Programmed Death-Ligand 1 (PD-L1) Blockade in Patients With Non-Small-Cell Lung Cancer Profiled With Targeted Next-Generation Sequencing. J Clin Oncol 2018;36:633–41. 10.1200/JCO.2017.75.3384 29337640 PMC6075848

[22] Takada K , Okamoto T , Toyokawa G , et al . The expression of PD-L1 protein as a prognostic factor in lung squamous cell carcinoma. Lung Cancer 2017;104:7–15. 10.1016/j.lungcan.2016.12.006 28213003

[23] Schroeder W , Ng L , Cates J . The ITK software guide, 2003.

[24] Bican J , Janeba D , Táborská K , et al . Image overlay using alpha-blending technique. Nucl Med Rev Cent East Eur 2002;5:53. 14600949

[25] Chollet F . Deep learning mit python und Keras: Das Praxis-Handbuch vom Entwickler der Keras-Bibliothek. MITP-Verlags GmbH & Co. KG, 2018.

[26] Grad-cam: Visual explanations from deep networks via gradient-based localization. Proceedings of the IEEE International Conference on computer vision 2017.

[27] DeLong ER , DeLong DM , Clarke-Pearson DL . Comparing the areas under two or more correlated receiver operating characteristic curves: a nonparametric approach. Biometrics 1988;44:837. 10.2307/2531595 3203132

[28] Rovere MTL , Bigger JT , Marcus FI , et al . Baroreflex sensitivity and heart-rate variability in prediction of total cardiac mortality after myocardial infarction. The Lancet 1998;351:478–84. 10.1016/S0140-6736(97)11144-8 9482439

[29] Diedenhofen B , Musch J . cocor: a comprehensive solution for the statistical comparison of correlations. PLoS One 2015;10:e0121945. 10.1371/journal.pone.0121945 25835001 PMC4383486

[30] Gainor JF , Shaw AT , Sequist LV , et al . EGFR mutations and ALK rearrangements are associated with low response rates to PD-1 pathway blockade in non-small cell lung cancer: a retrospective analysis. Clin Cancer Res 2016;22:4585–93. 10.1158/1078-0432.CCR-15-3101 27225694 PMC5026567

[31] Lambin P , Leijenaar RTH , Deist TM , et al . Radiomics: the bridge between medical imaging and personalized medicine. Nat Rev Clin Oncol 2017;14:749–62. 10.1038/nrclinonc.2017.141 28975929

[32] Chen R , Zhou X , Liu J , et al . Relationship between the expression of PD-1/PD-L1 and 18F-FDG uptake in bladder cancer. Eur J Nucl Med Mol Imaging 2019;46:848–54. 10.1007/s00259-018-4208-8 30627815

[33] Jiang M , Sun D , Guo Y , et al . Assessing PD-L1 expression level by radiomic features from PET/CT in nonsmall cell lung cancer patients: an initial result. Acad Radiol 2020;27:171–9. 10.1016/j.acra.2019.04.016 31147234

[34] Patil PD , Bera K , Vaidya P , et al . Correlation of radiomic features with PD-L1 expression in early stage non-small cell lung cancer (ES-NSCLC) to predict recurrence and overall survival (OS). J Clin Oncol 2018;36:e24247–e47. 10.1200/JCO.2018.36.15_suppl.e24247

[35] Yu Y , Zeng D , Ou Q , et al . Association of survival and immune-related biomarkers with immunotherapy in patients with non-small cell lung cancer: a meta-analysis and individual patient-level analysis. JAMA Netw Open 2019;2:e196879–e79. 10.1001/jamanetworkopen.2019.6879 31290993 PMC6625073

[36] He B , Dong D , She Y , Di Dong YS , Zhou C , et al . Predicting response to immunotherapy in advanced non-small-cell lung cancer using tumor mutational burden radiomic biomarker. J Immunother Cancer 2020;8:e000550. 10.1136/jitc-2020-000550 32636239 PMC7342823

[37] Fancello L , Gandini S , Pelicci PG , et al . Tumor mutational burden quantification from targeted gene panels: major advancements and challenges. J Immunother Cancer 2019;7:183. 10.1186/s40425-019-0647-4 31307554 PMC6631597

[38] Tunali I , Gray JE , Qi J , et al . Novel clinical and radiomic predictors of rapid disease progression phenotypes among lung cancer patients treated with immunotherapy: an early report. Lung Cancer 2019;129:75–9. 10.1016/j.lungcan.2019.01.010 30797495 PMC6450086

[39] Tunali I , Tan Y , Gray JE . Hypoxia-Related radiomics predict immunotherapy response: a multi-cohort study of NSCLC. bioRxiv.

[40] Trebeschi S , Drago SG , Birkbak NJ , et al . Predicting response to cancer immunotherapy using noninvasive radiomic biomarkers. Ann Oncol 2019;30:998–1004. 10.1093/annonc/mdz108 30895304 PMC6594459

[41] Sun R , Limkin EJ , Vakalopoulou M , et al . A radiomics approach to assess tumour-infiltrating CD8 cells and response to anti-PD-1 or anti-PD-L1 immunotherapy: an imaging biomarker, retrospective multicohort study. Lancet Oncol 2018;19:1180–91. 10.1016/S1470-2045(18)30413-3 30120041

[42] Semenza GL . Hypoxia-Inducible factors in physiology and medicine. Cell 2012;148:399–408. 10.1016/j.cell.2012.01.021 22304911 PMC3437543

[43] Chang C-H , Qiu J , O'Sullivan D , et al . Metabolic competition in the tumor microenvironment is a driver of cancer progression. Cell 2015;162:1229–41. 10.1016/j.cell.2015.08.016 26321679 PMC4864363

[44] Sun S , Fei X , Mao Y , et al . PD-1(+) immune cell infiltration inversely correlates with survival of operable breast cancer patients. Cancer Immunol Immunother 2014;63:395–406. 10.1007/s00262-014-1519-x 24514954 PMC11029035

[45] Rangachari D , VanderLaan PA , Shea M , et al . Correlation between Classic Driver Oncogene Mutations in EGFR, ALK, or ROS1 and 22C3-PD-L1 ≥50% Expression in Lung Adenocarcinoma. J Thorac Oncol 2017;12:878–83. 10.1016/j.jtho.2016.12.026 28104537 PMC5403565

[46] Enninga EAL , Harrington SM , Creedon DJ , et al . Immune checkpoint molecules soluble program death ligand 1 and galectin-9 are increased in pregnancy. Am J Reprod Immunol 2018;79:e12795. 10.1111/aji.12795 PMC5814874 29205636

[47] Velcheti V , Schalper KA , Carvajal DE , et al . Programmed death ligand-1 expression in non-small cell lung cancer. Lab Invest 2014;94:107–16. 10.1038/labinvest.2013.130 24217091 PMC6125250

[48] Caldwell C , Johnson CE , Balaji VN , et al . Identification and validation of a PD-L1 binding peptide for determination of PDL1 expression in tumors. Sci Rep 2017;7:1–11. 10.1038/s41598-017-10946-2 29057919 PMC5651871

[49] Hirsch FR , McElhinny A , Stanforth D , et al . PD-L1 immunohistochemistry assays for lung cancer: results from phase 1 of the blueprint PD-L1 IHC assay comparison project. J Thorac Oncol 2017;12:208–22. 10.1016/j.jtho.2016.11.2228 27913228

[50] Tsao MS , Kerr KM , Kockx M , et al . PD-L1 immunohistochemistry comparability study in real-life clinical samples: results of blueprint phase 2 project. J Thorac Oncol 2018;13:1302–11. 10.1016/j.jtho.2018.05.013 29800747 PMC8386299

