# Supplementary material for: Non-invasive measurement of PD-L1 status and prediction of immunotherapy response using deep learning of PET/CT images
Source: J Immunother Cancer. 2021 Jun 16;9(6):e002118. doi: 10.1136/jitc-2020-002118 (PMC8211060; doi:10.1136/jitc-2020-002118)
Supplement: Supplementary data [file jitc-2020-002118supp001.pdf]

Supplementary data

Supplementary Methods

Supplemental S1: Inclusion criteria for SPH and MCC cohorts

For PD-L1 prediction, patients with informed consent were accrued from the Shanghai Pulmonary Hospital (SPH), Shanghai, China, between January 2017 and June 2018 with following inclusion criteria were included: 1) histologically confirmed primary NSCLC; 2) pathological examination of PD-L1 status before any treatment; 3) PET/CT scans obtained within one month before biopsy (Bx) for immunohistochemistry (IHC) and no treatments was performed during this interval; 5) baseline clinical characteristics (including age, sex, stage, histology, and smoking history) and gene (EGFR, ALK and ROS1) mutation status were available. Based on these inclusion criteria, 400 patients were identified and subsequently assigned to a training cohort (SPH-training, N = 284) and an independent test cohort (SPH-test, N = 116). Using the same inclusion criteria, 85 NSCLC patients were accrued from H. Lee Moffitt Cancer Center & Research Institute (MCC), Tampa, FL and were used as external independent test cohort for prediction of PD-L1 expression (MCC-PD-L1 cohort).

For the distinct cohorts to predict patient response and outcomes, 128 patients were identified with histologically confirmed advanced stage (stage IIIB and IV) NSCLC who were treated with immunotherapy (anti-PD-L1 or anti-PD-1) between June 2011 and December 2017 at MCC using the following criteria: 1) PET/CT images were available during the interval (less than 6 months) of the last treatment (or diagnosis) and the start of immunotherapy; 2) no other treatment were performed during the interval; 3) follow-up time was greater than 6 months; and 4) no immune-related severe adverse events (Grade according to Common Terminology Criteria for Adverse Events (CTCAE)>=3[S1]) were observed or reported during treatment; 5) baseline clinical characteristics (including age, sex, stage, histology, Eastern Cooperative Oncology Group (ECOG) scale, brain metastasis status, and smoking history) and gene (EGFR, ALK and ROS1) mutation status were available. Using the same inclusion criteria, a prospective validation cohort was curated of 49 NSCLC patients who were treated with immunotherapy between January 2018 to June 2019.

For the external VA cohort to validate the DLS and the prognostic models, 35 patients with available PET/CT images from 72 patients with advanced stage NSCLC treated with immunotherapy (anti-PD-L1 or anti-PD-1) between July 2015 and February 2019 were identified according to the above criteria.

The progression of the distinct ICI-treated cohorts used to investigate the association of the DLS and clinical characteristics with the clinical outcome including DCB (PFS>6month[20]), PFS, and OS, was defined using Response Evaluation Criteria in Solid Tumors (RECIST1.1)[21]. For PFS, an event was defined as death or either

clinical or RECIST based progression of cancer and the data were right-censored at 6 years and 1.5 years for the retrospective and prospective cohorts, respectively. For OS, an event was defined as death and the data were right censored at 6 years and 1.5 years for the retrospective and prospective cohorts, respectively. Because we don’t have as much follow-up time for the prospective cohort, these two cohorts have different censoring values. The index date for both OS and PFS was the date of initiation of immunotherapy.

[S1]. Institute, N. C. Common terminology criteria for adverse events (CTCAE) v4. 0. ,2010.

Supplemental S2: PD-L1 expression by immunohistochemistry (IHC)

To ensure a reliable PD-L1 IHC score, all patients in this study underwent surgical resection or biopsy of the primary tumor using standardized protocol. To reduce the sampling artifact, the portion of the tumor specimen was carefully examined, and the portion with more malignant cells, less differentiated cells, and less hemorrhage was subjected to histopathological confirmation within 2 weeks after the ^{18}F-FDG PET/CT scan. Furthermore, routine IHC analysis was performed to determine PD-L1 expression in all the lesions of SPH cohort and MCC-PD-L1 cohort using the same antibody. For the SPH cohort, the platform of Dako Link 48 and the antibody of Dako 22C3 (Agilent Cat# GE00621-2, RRID:AB_2833074) were used for PD-L1 staining to quantify the presence of PD-L1. For the MCC-PD-L1 cohort, the PD-L1 22C3 mouse monoclonal antibody (Agilent Cat# GE00621-2, RRID:AB_2833074) purchased from Dako, was performed utilizing the Dako EnVision FLEX visualization system on the Dako Autostainer Link 48. To compensate for reader bias, all the staining results were reviewed and analyzed by 2 experienced pathologists who were blinded to each other’s scores and unaware of the patients’ clinical information. When there was discrepancy, the two pathologists would have a mutual discussion to reach a consensus.

Supplemental S3: Details of the training of the deep learning model

Details of the small-residual-convolutional-network

The SResCNN is similar to the Resnet50 but with fewer layers and smaller number of residual blocks, and the architecture was shown in supplemental Figure S1. The architecture was comprised with one convblock (including a 3 × 3 convolutional layer followed by a batch normalization layer and a rectified linear unit (ReLU) activation layer), 8 residual blocks (Resblock), and one fully connected layer. Finally, a softmax activation layer was connected to the last fully connected layer, which was used to yield the prediction probabilities of nodule

candidates. To prevent overfitting, one dropout layer with probability of 0.5 was added to the fully connected layers. Additionally, the model was optimized using the binary cross entropy loss function.

Preparation of the input images

After registration using ITK-SNAP, a square or an irregular box, which was close to the boundary of the tumor, was delineated manually in ITK software firstly by experienced nuclear medicine radiologist, and then the input regions of interest (ROIs) could be generated automatically after, dilation, resize and fusion to keep the entire tumor and its peripheral region were included (Supplemental Figure S3). For each slice of the tumor, the area of the smallest square mask (SSM) including the delineated region was regarded as the area of the tumor in this slice (Supplemental Figure S3B). Because of the big difference of the central slice and peripheral slices, only the slices with the area larger than the 30% of the maximum tumor cross-sectional area of this patient were regarded as valid input images and were used as the input of the deep learning model. The area here means the area of the smallest square including the delineated region (Supplemental Figure S3C). 10,650 ROIs were generated for training. In order to keep the training data had more balanced label, cubic spline interpolation of the adjacent two slices from the same patient with positive PD-L1 expression was used to generate new augmented slices on the condition that each patient was used with the same times, and finally 14,011 ROIs (6,722 PD-L1 positive and 7,289 PD-L1 negative) were used as the training dataset.

All ROIs were resized to the same size (64×64) using cubic spline interpolation and were standardized by z-score normalization before input to the model. As such, the input ROI was subtracted from the mean intensity value and divided by the standard deviation of the image intensity, before inputting to the deep learning model, to reduce the offset effect due to different equipment and different reconstruction parameters [S2].

Training of the SResCNN network

The training of the model focuses on the optimization of the parameters of the SResCNN model to build a relationship between PET/CT images and PD-L1 expression status (positive: 1 or negative: 0). We employed binary cross entropy as the loss function and the Adam optimizer with an initial learning rate = 0.0001, beta_1=0.9, beta_2=0.999. The learning rate was reduced by a factor of 5 if no improvement of the loss of the validation dataset was seen for a ‘patience’ number (n=50) [S3] of epochs. The batch size was set to 64. During the training, augmentation including width/height-shift, horizontal/vertical-flip, rotation and zoom were used to reduce overfitting. The training was stopped after waiting an additional 50 epochs since the validation loss stopped to degrade. The learning rate and batch size was determined with five-fold-cross validation under the patient level, and the combination that yielded the best average accuracy on the validation folds was chosen.

Application of the SResCNN network

The generated ROI-based hyper-image was input into the SResCNN model after z-score normalization, and a deeply learned score (DLS) representing the PD-L1 positivity could be yielded after a sequential activation of convolution and pooling layers. To develop a robust prediction, all valid slices of each patient were fed into the SResCNN model and the average DLSs with equal weight for each slice was regarded as the final PD-L1 positive probability of the tumor.

[S2]. Oh, Shu Lih, et al. Automated diagnosis of arrhythmia using combination of CNN and LSTM techniques with variable length heart beats. Computers in biology and medicine 102 (2018): 278-287.

[S3] COONEY, Ciaran; FOLLI, Raffaella; COYLE, Damien. Optimizing layers improves CNN generalization and transfer learning for imagined speech decoding from EEG. In: 2019 IEEE International Conference on Systems, Man and Cybernetics (SMC). IEEE, 2019: 1311-1316.

Supplemental S4: Correlation investigation between necrosis and DLS

First, necrosis in PET images was defined as an area of hypometabolism within the hypermetabolic tumor (i.e., classically a rim of hypermetabolism with a hypometabolic center)[S4]. Hypometabolism (necrosis) is defined as the region with SUV less than 42% of the maximum SUV[39]. Then, the ratio of necrosis to global lesion volume of the PET images (termed the necrosis-to-global volume ratio, NVR) was calculated and expressed as percentage to quantify the necrosis.

Thus, the relation between necrosis and the DLS could be investigated by calculating the Spearman's correlation, and linear regression between NVR and DLS, and only the cases with necrosis regions were included for this experiment.

[S4] Rakheja R, Makis W, Tulbah R, Skamene S, Holcroft C, Nahal A, et al. Necrosis on FDG PET/CT correlates with prognosis and mortality in sarcomas. Am J Roentgenol 2013;201:170-7

Supplemental S5: Radiomic quality score (RQS)

Radiomics is a rapidly maturing field in machine learning. To rigorously assess the quality of study design, Lambin et al. developed a 36-point "Radiomics Quality Score" (RQS) metric that evaluates 16 different key components[31]. The full list of criteria is described in **Supplemental Table S2**, which shows that the current study had a RQS of 22. To put this in perspective, a recent meta-analysis[S5] analyzed 77 radiomics publications

and documented that the mean ± S.D. RQS across all studies was 9.4 ± 5.6, indicating that the current study is in the upper 5 percentage of radiomics study designs.

[S5]. Park JE, Kim D, Kim HS et al. Quality of science and reporting of radiomics in oncologic studies: room for improvement according to radiomics quality score and TRIPOD statement. Eur Radiol 2019.

Supplementary Figures

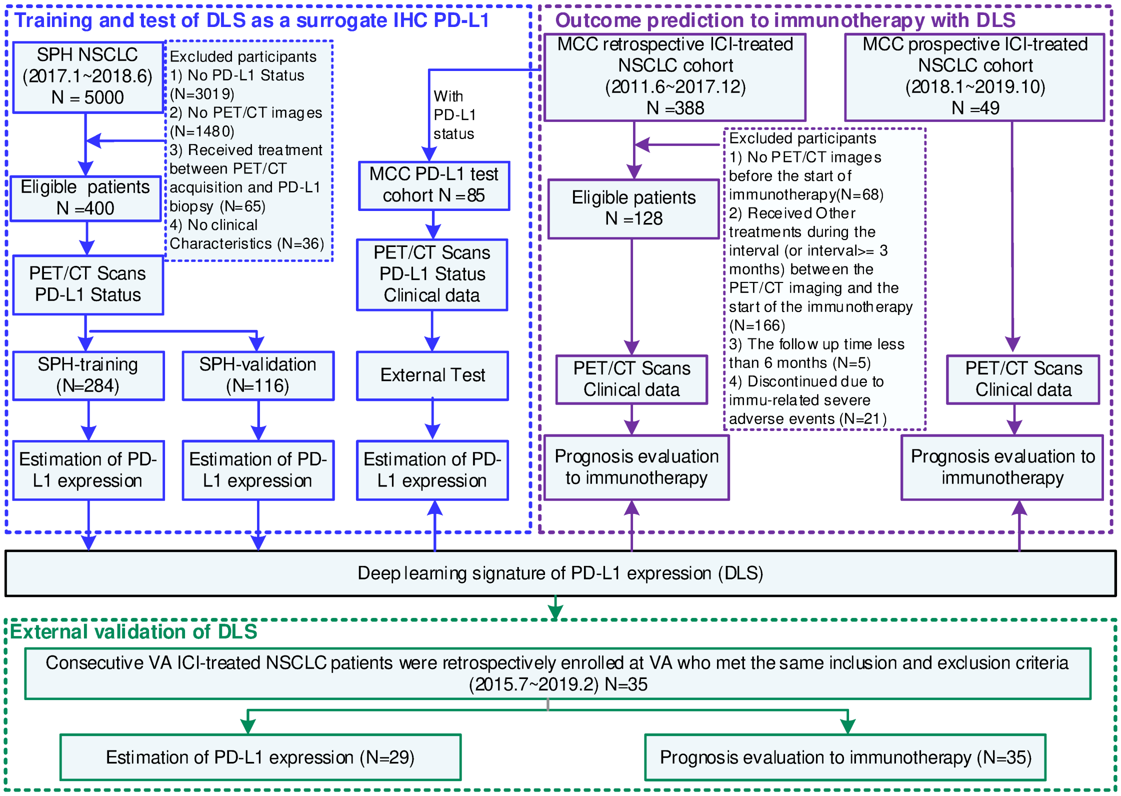

Figure S1. Study design and inclusion and exclusion diagram. The SPH data, which included PD-L1 expression data and the corresponding imaging data, was used to develop a deeply learned score (DLS) as a surrogate IHC based PD-L1 status. The MCC-PD-L1 data which included PD-L1 expression data, the corresponding imaging data and the treatment information was used for the clinical association and analytic validation analyses of the DLS through the measurement accuracy and the prognostic comparison, while the larger MCC retrospective and prospective cohorts comprised of patients treated with ICI were used for further clinical validation. Then the MCC ICI-treated retrospective and prospective cohorts were further used for developing and testing the prognosis prediction models that included other prognosis-related clinical variables. The external VA cohort was used as a further validation for PD-L1 status measurement and prognosis evaluation to ICI treatment.

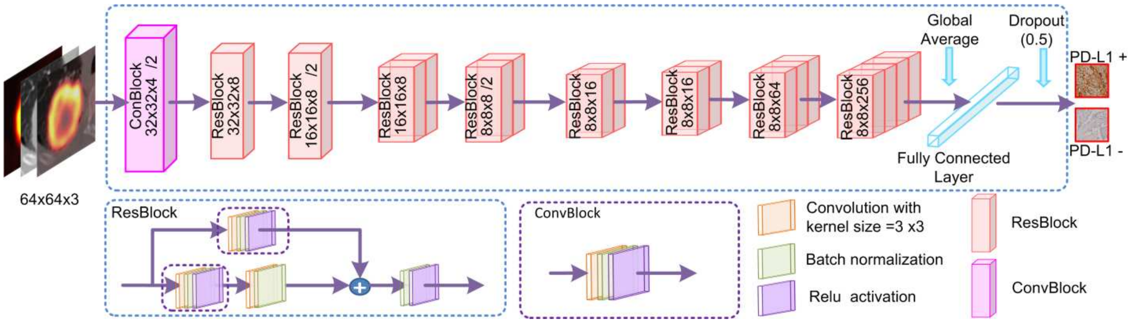

Figure S2. Illustration of the ResCNN model. This model is composed of convolutional layers with kernel size 3x3, batch normalization, pooling, and drop out layers. Note. /2 means the convolution layer of the Convblock with stride of 2**.**

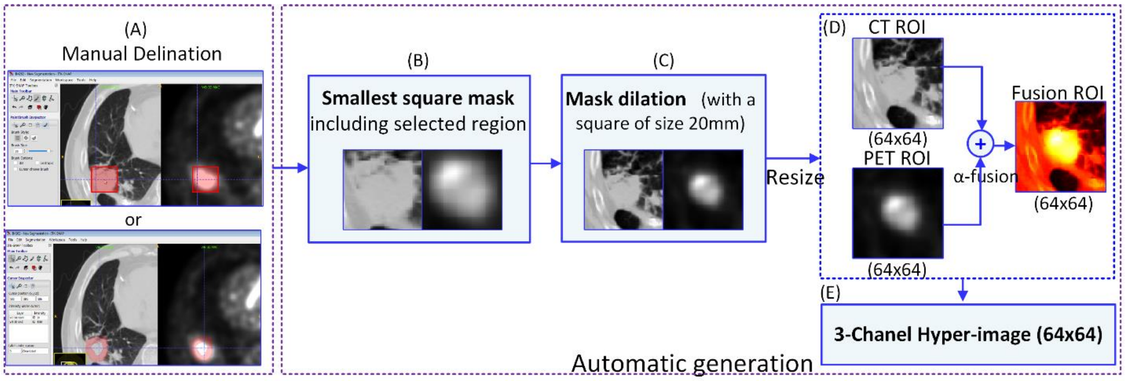

Figure S3. Illustration of the generation of the input hyper-image. A square or an irregular box, which was close to the boundary of the tumor, was delineated manually in ITK software firstly, and then the hyper-image was generated after dilation, resize and fusion automatically.

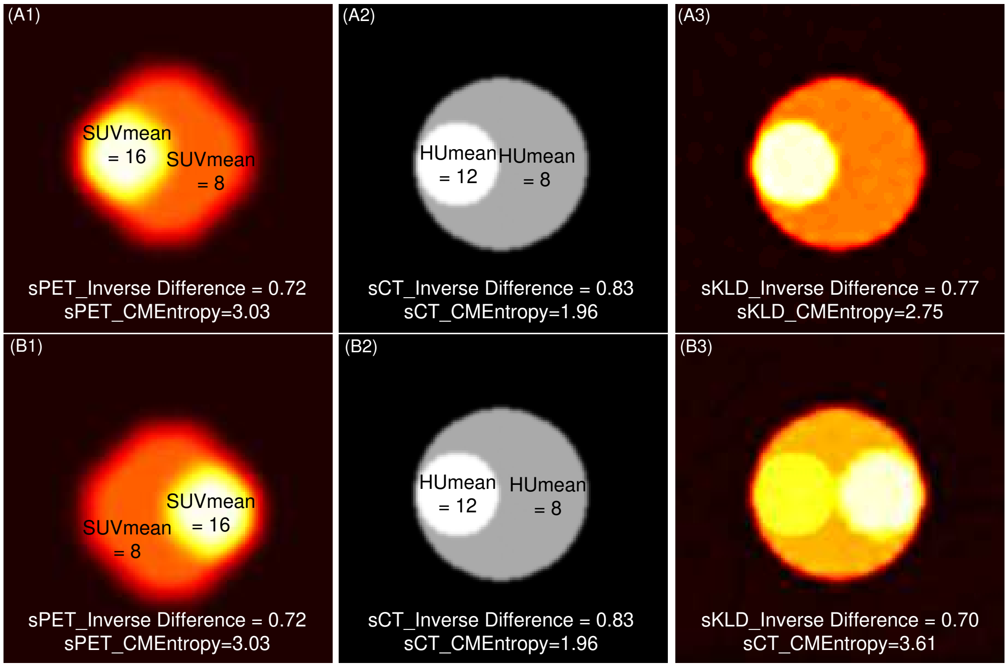

Figure S4. Two different digital simulated phantoms were constructed as A and B. To show the importance of the fusion images, A and B were kept to have the same heterogeneity distribution. Entropy and Inverse Difference calculated from 3D co-occurrence matrix were used to measure the heterogeneity and the homogeneity of the phantoms. From A1 and B1 (simulated PET images), A2 and B2 (simulated CT images), the two phantoms have the same heterogeneity and homogeneity distribution. But from A3 and B3, the two phantoms could be identified based on different heterogeneity and homogeneity, which means the fusion images could reflect the relative different positional relationship of the heterogeneity. Using this image to construct a 3-chanel hyper-image together with PET and CT images was more convenient for the training of the ResCNN model in one hand. In the other hand, incorporating the prior knowledge into the model can decrease the size of the deep learning model and limit the risk of overfitting.

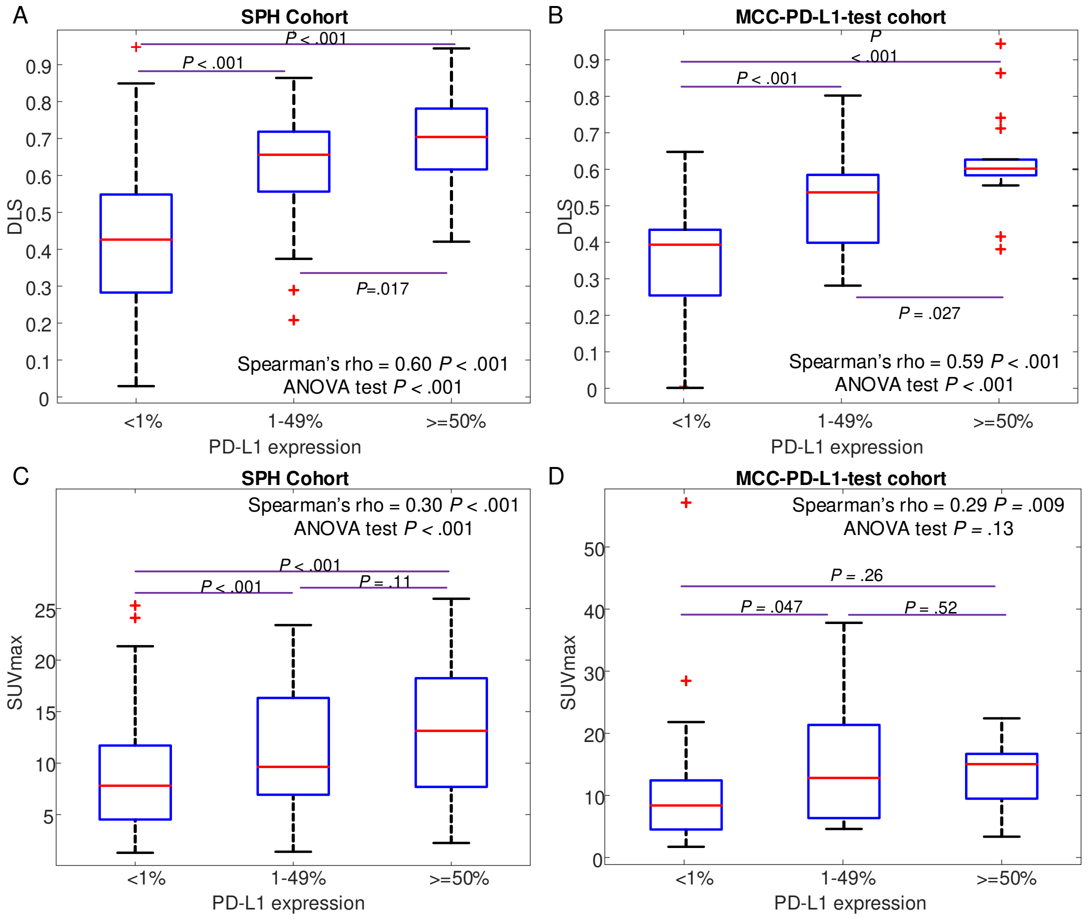

Figure S5. Distribution of the DLS and SUVmax across the PD-L1 expression. The p-values in the down (up)-right corner of each plot are from the Spearman's correlation analysis and ANOVA analysis. The *p*-values in the bridge between different cohorts are from the pairwise *post hoc* tests.

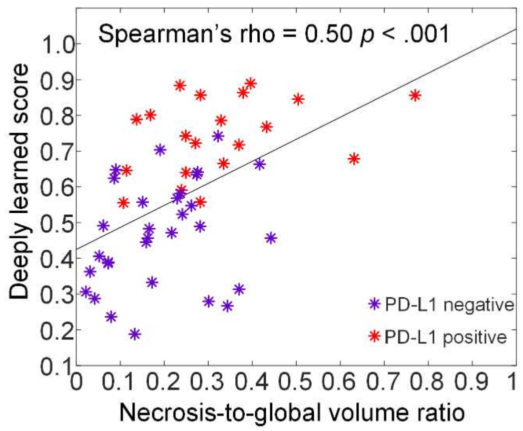

Figure S6. Correlation of the DLS and necrosis-to-global volume ratio within the patients possessing necrotic regions.

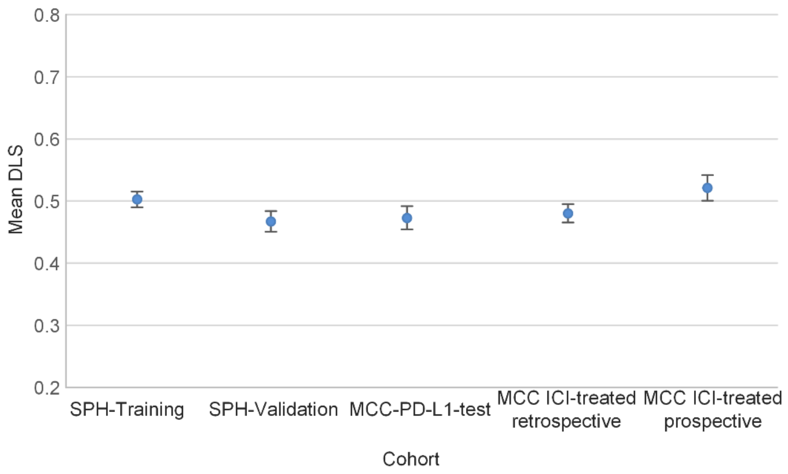

Figure S7. Distribution of the DLS across the patient cohorts. The bootstrapped mean value of DLSs as well as the standard error bar for different cohorts.

Mu W, *et al. J Immunother Cancer* 2021; 9:e002118. doi: 10.1136/jitc-2020-002118

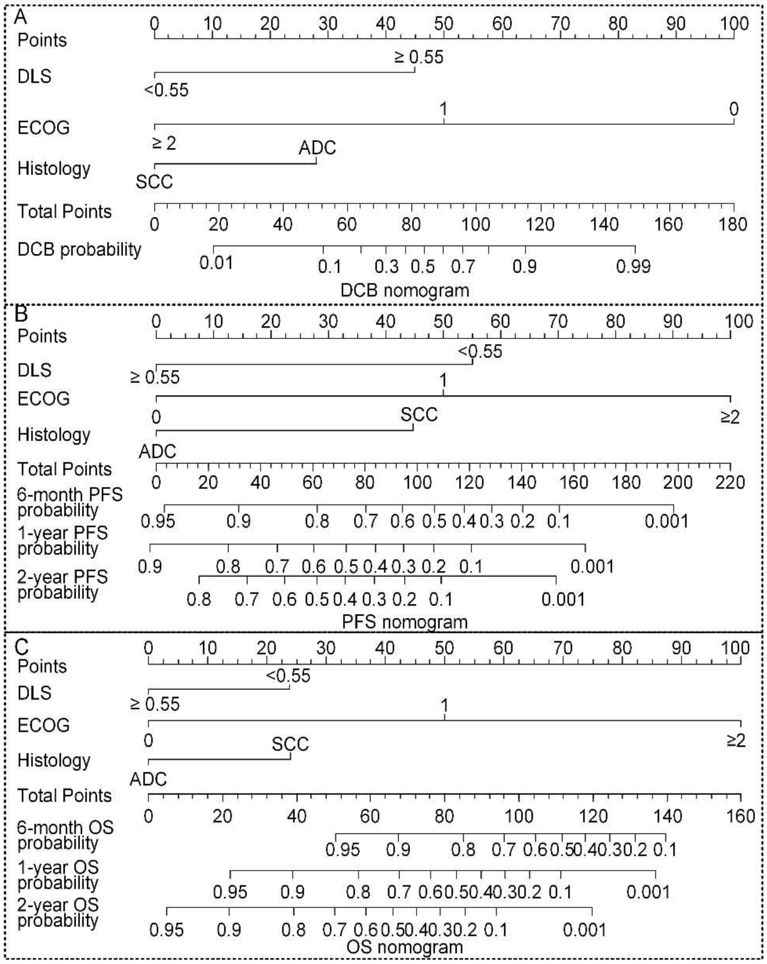

Figure S8. Nomograms for multivariable regression. (A) DCB nomogram obtained with multivariable logistic regression for DCB prediction (e.g., for a ADC patient with DLS of 0.6 and ECOG 1, his total points are 78 (DLS 0.6 corresponding to point 0, ECOG 1 corresponding to point 50, ADC corresponding to point 28, 0+50+28=78), which corresponds to a DCB probability of 0.40); (B) PFS nomogram obtained with multivariable Cox proportional hazards regression for PFS prediction (e.g., for a ADC patient with DLS of 0.6 and ECOG 1, his total points are 50 (DLS 0.6 corresponding to point 0, ECOG 1 corresponding to point 50, ADC corresponding to point 0, 0+50+0=50), which corresponds to a 6-month PFS probability of 0.85, 1-year PFS probability of 0.68, and 2-year PFS probability of 0.59). (C) OS nomograms obtained with multivariable Cox proportional hazards regression for OS prediction (e.g., for a SCC patient with DLS of 0.6 and ECOG 1, his total points are 74 (DLS 0.6 corresponding to point 0, ECOG 1 corresponding to point 50, SCC corresponding to point 24, 0+50+24=74), which corresponds to a 6-month OS probability of 0.87, 1-year OS probability of 0.63, and 2-year OS probability of 0.37).

Mu W, *et al. J Immunother Cancer* 2021; 9:e002118. doi: 10.1136/jitc-2020-002118

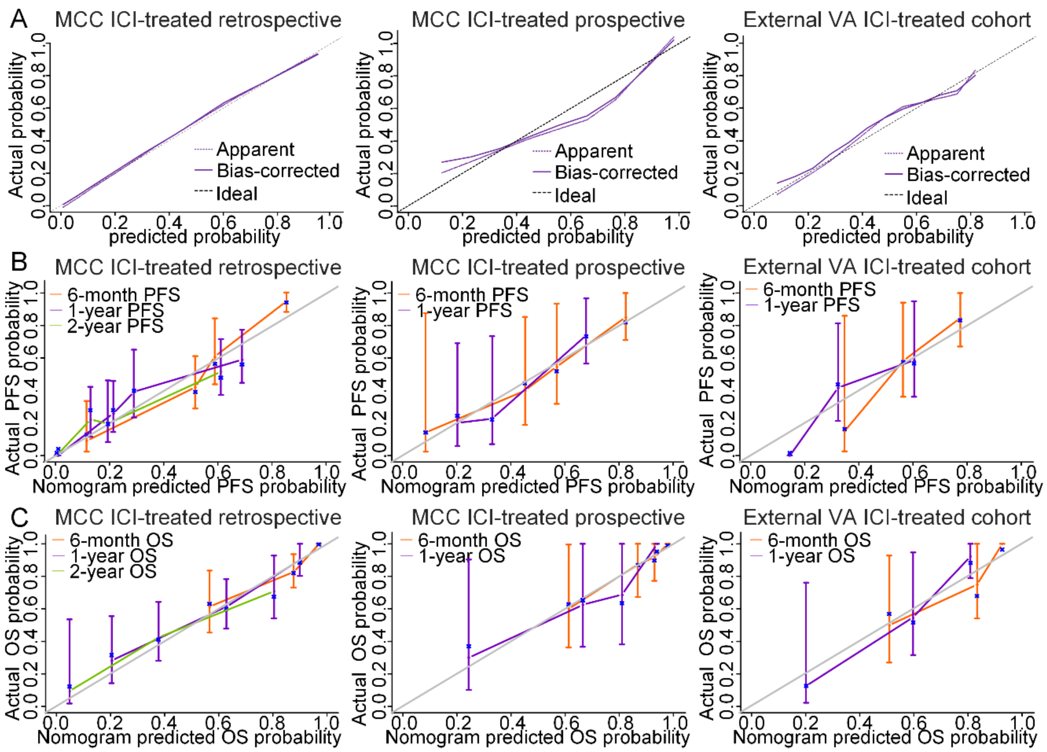

Figure S9. Calibration Plots for the multi-variable models. (A) The assessment of the DCB prediction model calibration in the MCC ICI-treated retrospective cohort (intercept = 0, slope = 0.99, C-index = 0.87), MCC ICI-treated prospective cohort (intercept = 0.067, slope = 0.73, C-index = 0.82), and the external VA ICI-treated cohort (intercept =-0.42, slope =0.61, C-index =0.81). (B) The assessment of the PFS prediction model calibration in the MCC ICI-treated retrospective cohort (6-month: slope = 1.00 [95%CI: 0.69-1.18], 1-year: slope = 1.00 [95%CI: 0.76-1.16], 2-year: slope = 1.00 [95%CI: 0.73-1.23] , C-index = 0.73), MCC ICI-treated prospective cohort (6-month: slope = 1.00 (95%CI: 0.28-1.45), 1-year: slope = 1.00 (95%CI: 0.09-1.37), C-index=0.74), and the external VA ICI-treated cohort (6-month: slope = 1.00 (95%CI: 0.40-1.79), 1-year: slope = 1.00 (95%CI: 0.07-1.78) , C-index=0.70). (C) The assessment of the OS prediction model calibration in the MCC ICI-treated retrospective cohort (6-month: slope = 1.00 [95%CI: 0.56-1.32], 1-year: slope = 1.00 [95%CI: 0.58-1.21],2-year: slope = 1.00 [95%CI: 0.67-1.21], C-index=0.74), MCC ICI-treated prospective cohort (6-month: slope = 1.00 [95%CI: -0.099-1.44], 1-year: slope = 1.00 [95%CI: 0.12-1.39] , C-index = 0.70), and the external VA ICI-treated cohort (6-month: slope = 1.00 [95%CI: 0.28-1.36], 1-year: slope = 1.00 [95%CI: 0.19-1.30] , C-index = 0.70) .

Supplementary Tables

Table S1. Acquisition parameters for the PET/CT imaging for each cohort

Characteristic	SPH	MCC-PD-L1 cohort	MCC retrospective ICI-treated cohort	MCC prospective ICI-treated cohort	VA cohort
Manufacturer, No. (%)[a]					
SIEMENS/CPS	400 (100)	64 (75.29)	19 (14.84)	12 (24.49)	35(100)
GE Medical	0	17 (20.00)	103 (80.47)	37 (75.51)	0
PHILIPS	0	4 (4.71)	6 (4.69)	0	0
Kilovoltage peak(kVp) , No. (%)					
120	400 (100)	77 (90.59)	118 (92.19)	44 (89.80)	35(100)
130	0	5 (5.88)	7 (5.47)	3 (6.12)	0
140	0	3 (3.53)	3 (2.34)	2 (4.08)	0
Reconstruction method, No. (%)					
OSEM	0	22 (25.88)	38 (21.69)	12 (24.49)	35(100)
PSF+TOF	400 (100)	4 (4.71)	7 (5.47)	2 (4.08)	0
VPHD	0	15 (17.65)	6 (4.69)	16 (32.65)	0
3D IR	0	43 (50.59)	73 (57.03)	19 (38.78)	0
'BLOB-OS-TF'	0	1 (1.18)	4 (3.13)	0	0
Current (mA)					
Median(range)	193 (90-463)	83 (31-238)	85 (27-299)	85 (29-299)	97(53-134)
Interval between administration and image acquisition					
Mean ± SD	62.68±12.13	95.39±18.84	96.26±24.03	96.06 ± 22.81	93.15±18.34
Dosage Mbq/kg					
Mean ± SD	3.70 ± 0.32	6.07 ± 2.11	6.03 ±1.87	5.97± 1.87	6.27±1.25
PET Slice Thickness					
Median(range)	5	3.27(3.26-5)	3.27(3.27-5)	3.27(3.26-5)	5
PET Pixel Spacing					
Median(range)	4.07	5.31(2.74-4.67)	5.47(2.73-4.67)	4.07(3.65-4.67)	4.07
CT Slice Thickness					
Median(range)	3	3.27(3.26-5)	3.375(3.27-5)	3.27(3.27-5)	2
CT Pixel Spacing					
Median(range)	0.9766	1.37(0.88-1.37)	1.37(0.88-1.37)	1.37(0.98-1.37)	0.9766

a. The PET/CT scaners of PHILIPS include GEMINI TF TOF16 and GEMINI TF TOF 16. The PET/CT scanners of SIEMENS include Biograph 6, Biograph 40, Biograph 64 and Emotion Duo. The PET/CT scanners of GE Medical include Discovery 600, Discovery STE and Discovery ST. The PET/CT scaners of CPS is 1080.

Mu W, *et al. J Immunother Cancer* 2021; 9:e002118. doi: 10.1136/jitc-2020-002118

Table S2. The criteria and maximal radiomic quality score as well as the actual score of this work

Criteria	Points system	Maximal score	Actual score of this work
Image protocol quality - well-documented image protocols (for example, contrast, slice thickness, energy, etc.) and/or usage of public image protocols allow reproducibility/replicability	+ 1 (if protocols are well-documented) + 1 (if public protocol is used)	2	1
Multiple segmentations - possible actions are: segmentation by different physicians/algorithms /software, perturbing segmentations by (random) noise, segmentation at different breathing cycles. Analyse feature robustness to segmentation variabilities	1	1	0
Phantom study on all scanners - detect inter-scanner differences and vendor-dependent features. Analyse feature robustness to these sources of variability	1	1	0
Imaging at multiple time points - collect images of individuals at additional time points. Analyse feature robustness to temporal variabilities (for example, organ movement, organ expansion /shrinkage)	1	1	0
Feature reduction or adjustment for multiple testing - decreases the risk of overfitting. Overfitting is inevitable if the number of features exceeds the number of samples. Consider feature robustness when selecting features	− 3 (if neither measure is implemented) + 3 (if either measure is implemented)	3	0
Multivariable analysis with non radiomics features (for example, EGFR mutation) - is expected to provide a more holistic model. Permits correlating /inferencing between radiomics and non radiomics features	1	1	1

Detect and discuss biological correlates - demonstration of phenotypic differences (possibly associated with underlying gene–protein expression patterns) deepens understanding of radiomics and biology	1	1	1
Cut-off analyses - determine risk groups by either the median, a previously published cut-off or report a continuous risk variable. Reduces the risk of reporting overly optimistic results	1	1	1
Discrimination statistics - report discrimination statistics (for example, C-statistic, ROC curve, AUC) and their statistical significance (for example, p-values, confidence intervals). One can also apply resampling method (for example, bootstrapping, cross-validation)	+ 1 (if a discrimination statistic and its statistical significance are reported) + 1 (if a resampling method technique is also applied)	2	2
Calibration statistics - report calibration statistics (for example, Calibration-in-the-large/slope, calibration plots) and their statistical significance (for example, P-values, confidence intervals). One can also apply resampling method (for example, bootstrapping, cross-validation)	+ 1 (if a calibration statistic and its statistical significance are reported) + 1 (if a resampling method technique is also applied)	2	0
Prospective study registered in a trial database - provides the highest level of evidence supporting the clinical validity and usefulness of the radiomics biomarker	+ 7 (for prospective validation of a radiomics signature in an appropriate trial)	7	7
Validation - the validation is performed without retraining and without adaptation of the cut-off value, provides crucial information with regard to credible clinical performance	- 5 (if validation is missing) + 2 (if validation is based on a dataset from the same institute) + 3 (if validation is based on a dataset from another institute) + 4 (if validation is based on two datasets from two distinct institutes) + 4 (if the study validates a previously published signature) + 5 (if validation is based on three or more datasets from distinct institutes)	5	4

Comparison to 'gold standard' - assess the extent to which the model agrees with/is superior to the current 'gold standard' method (for example, TNM-staging for survival prediction). This comparison shows the added value of radiomics	2	2	2
Potential clinical utility - report on the current and potential application of the model in a clinical setting (for example, decision curve analysis)	2	2	0
Cost-effectiveness analysis - report on the cost-effectiveness of the clinical application (for example, QALYs generated)	1	1	0
Open science and data - make code and data publicly available. Open science facilitates knowledge transfer and reproducibility of the study	+ 1 (if scans are open source) +1 (if region of interest segmentations are open source) + 1 (if code is open source) + 1 (if radiomics features are calculated on a set of representative ROIs and the calculated features and representative ROIs are open source)	4	3
Total score		36	22

Table S3. Demographic and clinical characteristics of VA ICI-treated patients

Characteristic	All (N=35)	Deep Learning Score		*P*
		High (N=28)	Low (N=7)	
Age(y)				0.76
Mean ± SD	71.40±7.19	71.77±7.36	71.29±9.05	
Sex, NO. (%)				NaN
Male	35 (100)	26 (100)	7 (100)	
Female	0	0	0	
TNM stage				0.64
III	10 (28.57)	9 (31.03)	1 (16.67)	
IV	25 (71.43)	19 (68.97)	6 (83.33)	
Histology (baseline), NO. (%)				0.19
ADC	19 (54.29)	14 (50.00)	5 (83.33)	
SCC	16 (45.71)	14 (51.72)	2 (16.67)	
EGFR, NO. (%)				NaN
Mutation	0	0	0	
Wild	22 (62.86)	17 (58.62)	5 (83.33)	
ALK, NO. (%)				0.74
Mutation	1 (2.86)	1 (3.45)	0	
Wild	7 (20.00)	5(17.24)	2 (33.33)	
ROS1, NO. (%)				NaN
Mutation	0	0	0	
Wild	22 (62.86)	17 (58.62)	5 (83.33)	
Smoke, NO. (%)				1.00
Never	1 (2.86)	1 (3.45)	0	
Former	34 (97.14)	27(96.55)	9 (100)	
ECOG Scale, NO. (%)				0.42
0	7 (20.00)	5 (17.24)	2 (33.33)	
1	22 (62.86)	18 (62.07)	4 (66.67)	
>=2	6 (17.14)	5 (20.69)	1	
Clinical Benefit, NO. (%)				0.032
DCB	19 (54.29)	18 (62.07)	1 (16.67)	
NDB	16 (45.71)	10 (37.93)	6 (83.33)	
Progression-free Survival				0.038*
Median (95%CI)	8.13(4.50,11.77)	9.30 (4.69,13.91)	2.37 (1.60,3.13)	
Overall Survival				0.007*
median (95%CI)	13.10 (5.67,20.53)	15.53 (9.54,21.53)	4.93 (2.20,7.67)	
PD-L1 by IHC				0.007*
Positive	24 (68.57)	20 (72.41)	4 (50.00)	
Negative	5 (14.29)	2 (6.90)	3 (50.00)	
Deep Learning Score				<.001*
Median(IQR)	0.63(0.56,0.66)	0.64 (0.58,0.67)	0.37 (0.28,0.46)	

Note. PD-L1 expression status was significantly associated with DCB (*p*=0.017, Fisher's Exact Test).

Table S4. Predictive performance of the ResCNN model by histology subtypes

AUC [95%CI]	SPH-training cohort	SPH-validation cohort	MCC-PD-L1-test cohort	External VA PD-L1 test cohort
DLS				
ADC	0.89 [0.84,0.94]	0.81 [0.71,0.91]	0.88 [0.76, 0.96]	0.89 [0.64, 1.00]
SCC	0.87 [0.80,0.95]	0.89 [0.76,1.00]	0.77 [0.61,0.91]	0.80 [0.50, 1.00]
ADC + SCC	0.89 [0.85,0.93]	0.84 [0.76,0.92]	0.82 [0.74-0.89]	0.82 [0.65,0.98]
SUVmax				
ADC	0.66 [0.57, 0.74]	0.72 [0.59, 0.84]	0.63 [0.44,0.79]	0.68 [0.43,0.93]
SCC	0.67 [0.54. 0.79]	0.41 [0.20, 0.61]	0.71 [0.53,0.86]	0.47 [0,0.90]
ADC + SCC	0.69 [0.62, 0.75]	0.68 [0.57, 0.78]	0.66 [0.53,0.77]	0.56 [0.28,0.84]

Accuracy [95%CI]	SPH-training cohort	SPH-validation cohort	MCC-PD-L1-test cohort	External VA PD-L1 test cohort
DLS				
ADC	83.33[78.20,88.23]	79.76[71.43,87.47]	82.98[70.34,93.62]	81.25[62.50,100]
SCC	77.50[67.50,86.25]	75.00[59.38,87.50]	71.05[55.26,84.21]	76.92[53.85,92.31]
ADC + SCC	81.69[77.11,85.91]	78.45[71.55,85.3]	77.65[69.41,85.88]	79.31[65.52,93.10]
SUVmax				
ADC	75[69.12,79.41]	60.71[50.00,71.43]	59.57[44.68,72.34]	68.75[37.50,81.25]
SCC	60[50,71.25]	43.75[28.12,62.5]	57.89[42.11,71.05]	53.85[30.77,76.92]
ADC + SCC	70.77[64.79,75.35]	67.24[59.48,75]	62.35[47.06,68.24]	55.83[34.48,68.97]

Sensitivity [95%CI]	SPH-training cohort	SPH-validation cohort	MCC-PD-L1-test cohort	External VA VA PD-L1 test cohort
DLS				
ADC	83.33[70.83,93.75]	63.16[42.11,84.21]	73.08[55.87,88.46]	78.57[57.14,100]
SCC	86.49[75.68,94.59]	81.25[56.25,100]	63.64[40.91,81.82]	90[70,100]
ADC + SCC	84.71[76.47,91.76]	77.43[57.14,85.71]	68.75[55.26,81.25]	83.33[66.67,95.83]
SUVmax				
ADC	72.92[31.25,95.83]	68.42[47.37,89.47]	69.23[50,84.62]	64.29[28.57,78.57]
SCC	67.57[35.14,78.38]	37.50[12.50,62.50]	50.00[31.82,68.18]	50.00[20.00,80.00]
ADC + SCC	52.94[35.29,85.88]	42.86[28.57,60.00]	60.42[47.06,68.23]	45.83[29.17,62.50]

	SPH-training cohort	SPH-validation cohort	MCC-PD-L1-test cohort	External VA PD-L1 test cohort
DLS				
ADC	83.33[77.56,89.10]	84.62[75.38,92.31]	95.24[85.71,100]	100
SCC	69.77[55.81,81.40]	68.75[50.00,87.50]	81.25[62.50,100]	33.33[0,100]
ADC + SCC	80.40[74.87,85.67]	81.48[72.84,88.89]	89.19[78.38,97.30]	60.00[20.00,100]
SUVmax				
ADC	51.92[43.59,58.97]	58.46[46.15,69.96]	47.62[23.81,71.43]	100.00[0,100]
SCC	72.09[58.14,86.05]	50.00[12.50,62.50]	68.75[43.75,87.50]	66.67[0,100]
ADC + SCC	78.39[72.86,83.92]	77.78[69.14,86.42]	64.86[48.65,81.08]	80.00[40.00,100]

Note. Cutoff for DLS is 0.55 for ADC cohort, SCC cohort and ADC+SCC cohort for all three cohorts. Cutoffs for SUV are 6.8, 14.5 and 12.11 for ADC cohort, SCC cohort and ADC+SCC cohort for all three cohorts, respectively, according to the ROC curves of training cohort.

Table S5. Univariable analysis of risk factors for DCB prediction

	**Retrospective**		**Prospective**	
	Odds Ratio (95% CI)	p	Odds Ratio (95% CI)	P
Age	1.02 (0.99-1.05)	0.11	0.97 (0.92-1.04)	0.39
BMI	1.13 (1.04-1.22)	0.002	0.87 (0.76-1.00)	0.051
Sex	0.86 (0.43-1.70)	0.66	1.62 (0.49-5.32)	0.43
Stage	0.74 (0.30-1.79)	0.50	-	-
Brain Metastasis	0.20 (0.02-1.86)	0.16	1.50 (0.42-5.32)	0.53
Histology(baseline)	0.39 (0.19-0.82)	0.013	0.06 (0.013-0.27)	<.001
EGFR	0.74 (0.17-3.14)	0.68	-	-
ALK	0.75 (0.045-12.30)	0.84	-	-
ROS1	-	-	-	-
Smoking status	0.54 (0.26-1.12)	0.096	1.64 (0.46-5.87)	0.45
ECOG	0.055(0.012-0.24)	<.001	0.77 (0.17-3.44)	0.73
SUVmax	1.01 (0.96-1.06)	0.74	1.11 (1.00-1.23)	.047

Note., For Sex: male was assigned 1 and female was assigned 2; for histology, ADC was assigned 1 and SCC was assigned 2.

Table S6. Univariable analysis of risk factors for PFS

	**Retrospective**		**Prospective**	
	Hazard ratio (95% CI)	p	Hazard ratio (95% CI)	p
Age	0.99(0.98-1.01)	0.53	1.03(0.99-1.07)	0.21
BMI	0.96 (0.92-1.00)	0.048	1.05 (0.96-1.15)	0.25
Sex	0.97 (0.65-1.44)	0.86	0.76 (0.36-1.62)	0.48
Stage	1.04 (0.62-1.72)	0.89	1.99 (0.59-6.71)	0.27
Brain Metastasis	1.55 (0.5-4.25)	0.40	0.62 (0.22-1.8)	0.38
Histology(baseline)	2.13 (1.40-3.24)	<.001	6.22 (2.70-14.308)	<.001
EGFR	0.49 (0.15-1.56)	0.23	0.043 (0-41.73)	0.37
ALK	1.31 (0.32-5.36)	0.71	-	-
ROS1	0.53 (0.07-4.05)	0.54	-	-
Smoking status	1.04 (0.67-1.60)	0.88	0.95(0.42-2.15)	0.89
ECOG	2.40 (1.38-4.19)	0.002	0.82 (0.35-1.95)	0.66
SUVmax	1.00 (0.97-1.03)	0.97	0.99 (0.95-1.03)	0.62

Note., For Sex: male was assigned 1 and female was assigned 2; for histology, ADC was assigned 1 and SCC was assigned 2.

Mu W, *et al. J Immunother Cancer* 2021; 9:e002118. doi: 10.1136/jitc-2020-002118

Table S7. Univariable analysis of risk factors for OS

	Retrospective		Prospective	
	Hazard ratio (95% CI)	p	Hazard ratio (95% CI)	p
Age	0.99 (0.97-1.01)	0.31	1.01 (0.96-1.07)	0.74
BMI	0.93 (0.88-0.99)	0.017	1.01 (0.91-1.13)	0.80
Sex	0.87(0.51-1.49)	0.87	0.86 (0.29-2.57)	0.79
Stage	1.24 (0.60-2.56)	0.56	1.29 (0.27-6.09)	0.75
Brain Metastasis	2.17 (0.67-7.01)	0.20	1.26 (0.34-4.63)	0.73
Histology(baseline)	2.44(1.38-4.30)	0.002	5.34 (1.40-20.32)	0.014
EGFR	1.09 (0.26-4.59)	0.91	0.043 (0-1950)	0.57
ALK	0.045 (0-68.25)	0.41	-	-
ROS1	21.10 (0-Inf)	0.77	-	-
Smoking status	1.64 (0.72-2.50)	0.35	1.11 (0.34-3.64)	0.87
ECOG	6.24 (1.94-20.11)	0.002	0.75 (0.23-2.47)	0.63
SUVmax	1.01(0.97-1.04)	0.74	0.97 (0.90-1.05)	0.48

Note., For Sex: male was assigned 1 and female was assigned 2; for histology, ADC was assigned 1 and SCC was assigned 2.

Table S8. Clinical characteristics associated with patient outcomes

Characteristic	K-M Anaylsis				Cox regression			
	MCC retrospective ICI cohort		MCC prospective ICI cohort		MCC retrospective ICI cohort		MCC prospective ICI cohort	
	median time (IQR), months	*p*	median time (IQR), months	*p*	HR [95%CI]	*p*	HR [95%CI]	*p*
**Histology**								
PFS								
ADC<br>SCC	10.60 [3.73,50.20]<br>3.93 [1.63, 8.40]	<.001*	17.00 [7.93, NR]<br>4.00 [3.00, 5.73]	<.001*	2.13 [1.40, 3.24]	<.001*	6.22 [2.70,14.31]	<.001*
OS								
ADC<br>SCC	NR [12.13, NR]<br>11.07 [6.47, 27.60]	0.002*	NR [17.00, NR]<br>11.43 [11.23, NR]	.006*	2.44 [1.38, 4.30]	.002*	5.34 [1.40, 20.32]	.014*
**ECOG**								
PFS								
0<br>>=1	12.47 [7.67, NR]<br>5.50 [0.3, 15.80]	.001*	8.37 [5.76, 17.00]<br>7.93 [3.93, -]	0.65	2.40 [1.38,4.20]	.002*	0.82 [0.35, 1.95]	0.66
OS								
0<br>>=1	NR [23.87, NR]<br>15.10 [6.57, NR]	<.001*	17.00 [11.23, NR]<br>NR [11.43, NR]	0.63	6.24 [1.94, 20.11]	.002*	0.75 [0.23, 2.47]	0.63

Note: NR means the median (or 25%, or 75%) of survival has been not yet reached.

Table S9. DLS and patient outcomes stratified by histology subtypes

Histology	MCC retrospective IO cohort				MCC prospective IO cohort			
	ADC		SCC		ADC		SCC	
	High DLS (N=23)	Low DLS (N=57)	High DLS (N=20)	Low DLS (N=28)	High DLS (N=20)	Low DLS (N=8)	High DLS (N=11)	Low DLS (N=10)
PFS								
HR [95%CI]	0.26 [0.12,0.58]		0.42 [0.22, 0.81]		0.30 [0.093,0.97]		0.54 [0.18,1.61]	
p (cox)	0.001*		0.010*		0.045*		0.27	
p (K-M)	<0.001*		0.008*		0.034*		0.25*	
OS								
HR [95%CI]	0.38 [0.13,1.12]		0.48 [0.21,1.06]		0.087 [0.008,0.90]		0.29 [0.058,1.44]	
p (cox)	0.080		0.068		0.041*		0.13	
p (K-M)	0.069		0.061*		0.007*		0.10	
Durable Clincial benefit								
DCB rate	91.30%	50.88%	65.00%	21.43%	100.00%	62.50%	45.45%	20.00%
ORR[95%CI]	10.14 [2.17,47.32]		6.81 [1.88,24.69]		-		3.33 [0.47, 23.47]	
p(logistic)	0.003*		0.004*		-		0.23	

Note., : * means P value <.05

Table S10. DLS and clinical outcomes stratified by ECOG performance status

ECOG	MCC retrospective IO cohort				MCC prospective IO cohort			
	ECOG=0		ECOG>=1		ECOG=0		ECOG>=1	
	High DLS (N=7)	Low DLS (N=22)	High DLS (N=36)	Low DLS (N=63)	High DLS (N=5)	Low DLS (N=5)	High DLS (N=26)	Low DLS (N=13)
PFS								
HR [95%CI]	0.15 [0.019, 1.14]		0.38 [0.23,0.64]		0.37 [0.11,2.73]		0.22 [0.09, 0.55]	
p (cox)	0.066		<.001*		0.25		0.001*	
p (K-M)	0.025		<0.001*		0.21		<.001*	
OS								
HR[95%CI]	0.019 [0.00,229.61]		0.45 [0.24, 0.87]		0.78 [0.069,8.88]		0.048 [0.006,0.40]	
p (cox)	0.41		0.009*		0.84		0.005*	
p (K-M)	0.12		0.014*		0.83		<.001*	
Durable Clincial benefit								
DCB rate	100%	90.91%	74.29%	23.81%	80.00%	60.00%	80.77%	30.77%
ORR[95%CI]	-		9.60 [3.71,24.86]		2.67 [0.16,45.14]		9.45 [2.05,43.61]	
p(logistic)	-		<.001		0.50		0.004	

Note., : * means P value <.05

Mu W, *et al. J Immunother Cancer* 2021; 9:e002118. doi: 10.1136/jitc-2020-002118

Table S11. Multivariable logistic regression and Cox regression analyses

	DCB prediction			PFS estimation			OS estimation		
	B	Odds Ratio (95% CI)	*p*	B	Hazard Ratio (95% CI)	*p*	B	Hazard Ratio (95% CI)	*p*
DLS	3.00	20.13 (5.71-71.00)	<.001	-1.37	0.25 (0.15-0.42)	.002	-1.01	0.36 (0.19-0.69)	0.002
Histology	-1.91	0.15 (0.045-0.49)	<.001	1.12	3.06 (1.95-4.80)	<.001	1.03	2.79(1.58-4.95)	<.001
ECOG	-3.41	0.033 (0.008-0.14)	<.001	1.26	3.52(2.17-5.69)	<.001	2.13	8.37 (3.91-17.92)	<.001
Constant	4.54		<.001						
C-Index (95%CI, p-value)									
Retrospective		0.87 (0.82-0.92, <.001)			0.73 (0.68-0.78, <.001)			0.77 (0.71-0.84, <.001)	
Prospective		0.84 (0.74-0.94, <.001)			0.74 (0.67-0.87, <.001)			0.70 (0.50-0.87, 0.02)	
VA		0.81 (0.70-0.93, <.001)			0.70 (0.59-0.80, <.001)			0.70 (0.59-0.81, <0.001)	
AIC									
Retrospective		118.12			703.23			371.19	
Prospective		49.58			173.36			83.57	
VA		40.50			130.16			105.75	

Table S12. Multivariable logistic regression and Cox regression analysis only using clinical characteristics

	DCB prediction			PFS estimation			OS estimation		
	B	Odds Ratio (95% CI)	*p*	B	Hazard Ratio (95% CI)	*p*	B	Hazard Ratio (95% CI)	*p*
ECOG	-2.54	0.079 (0.023-0.27)	<.001	0.98	2.68(1.65-4.34)	<.001	2.11	8.11 (3.73-17.65)	<.001
Histology	-0.96	0.38 (0.17-0.87)	.023	0.79	2.20(1.44-3.37)	<.001	0.95	2.58 (1.46-4.56)	0.001
Constant	3.71		<.001						
C-Index (95%CI, p-value)									
Retrospective		0.75 (0.69-0.81, <.001)			0.67 (0.62-0.72, <.001)			0.74 (0.67-0.81, <.001)	
Prospective		0.72 (0.60-0.85, 0.004)			0.61 (0.50-0.72, 0.049)			0.60 (0.44-0.76, 0.24)	
VA		0.70 (0.57-0.84, 0.014)			0.61 (0.50-0.72, 0.052)			0.67 (0.54-0.79, 0.008)	
AIC									
Retrospective		149.73			733.37			379.75	
Prospective		62.50			188.03			87.57	
VA		46.65			134.27			109.04	
P value of Z-test compared with the models in Table S11									
Retrospective		<.001			0.024			0.001	
Prospective		0.050			0.009			0.001	
VA		0.029			0.076			0.036	

Mu W, *et al. J Immunother Cancer* 2021; 9:e002118. doi: 10.1136/jitc-2020-002118
